# Effectiveness of information and communication technology-based integrated care for older adults: a systematic review and meta-analysis

**DOI:** 10.3389/fpubh.2023.1276574

**Published:** 2024-01-05

**Authors:** Yutong Tian, Shanshan Wang, Yan Zhang, Lixue Meng, Xiaohua Li

**Affiliations:** ^1^School of Nursing and Health, Zhengzhou University, Zhengzhou, Henan, China; ^2^School of Nursing, The Hong Kong Polytechnic University, Hong Kong, China

**Keywords:** information and communication technology, integrated care, older adults, systematic review, meta-analysis

## Abstract

**Background:**

Information and communication technology (ICT) is a key factor in advancing the implementation of integrated care for older adults in the context of an aging society and the normalization of epidemics. This systematic review aims to comprehensively evaluate the effectiveness of ICT-based integrated care for older adults to provide input for the construction of intelligent integrated care models suitable for the context of an aging population in China.

**Methods:**

A systematic review and meta-analysis were conducted using PubMed, Web of Science Core Collection, Scopus, MEDLINE, EBSCO, EMBASE, CINAHL with full text, ProQuest, and Cochrane Library databases, along with the Google Scholar search engine, for papers published between January 1, 2000, and July 25, 2022, to include randomized controlled trials and quasi-experimental studies of ICT-based integrated care for older adults. Two reviewers independently performed literature screening, quality assessment (JBI standardized critical appraisal tool), and data extraction. The results were pooled using a random effects model, and narrative synthesis was used for studies with insufficient outcome data.

**Results:**

We included 32 studies (21 interventions) with a total of 30,200 participants (14,289 in the control group and 15,911 in the intervention group). However, the quality of the literature could be improved. The meta-analysis results showed that ICT-based integrated care significantly improved the overall perceived health status of older adults (n=3 studies, MD 1.29 (CI 0.11 to 2.46), no heterogeneity) and reduced the number of emergency department visits (n=11 studies, OR 0.46 (CI 0.25 to 0.86), high heterogeneity) but had no significant effect on improving quality of life, mobility, depression, hospital admissions and readmissions, or mortality in older adults, with a high degree of study heterogeneity. Narrative analysis showed that the overall quality of care, primary care service use, and functional status of older adults in the intervention group improved, but the cost-effectiveness was unclear.

**Conclusions:**

ICT-based integrated care is effective in improving health outcomes for older adults, but the quality and homogeneity of the evidence base need to be improved. Researchers should develop intelligent integrated care programs in the context of local health and care welfare provision systems for older adults, along with the preferences and priorities of the older adults.

## 1 Introduction

The World Health Organization (WHO) has proposed integrated care as a future approach to strengthening primary health care and coordinating health and social care as a proactive response to population aging since the 1990s. Integrated care refers to providing care throughout the whole life cycle, including continuity and coordination of health promotion, disease prevention, and treatment and rehabilitation through management and services that are person-centered, comprehensive, and multidisciplinary (1). The plan for the Decade of Healthy Aging (2020–2030), approved by the World Health Assembly in August 2020, specifically calls for the implementation of integrated care (2). The WHO has also developed an evidence-based Integrated Care for Older Adults Program that aims to manage the decline of intrinsic capacity and to promote integration and interaction between older adults and their environment to implement the Decade of Healthy Aging and ICT-based integrated care (3).

ICT includes any communication device or application that stores, retrieves, manipulates, transmits, or receives electronic information in digital form (e.g., telephone, computer, television, email, or robot) with the advantage of sharing information across professional and organizational boundaries (4, 5). ICT provides a platform for patient empowerment, condition monitoring, and self-management, and has been identified as an important enabler for the provision of integrated and coordinated primary health care services (6). The WHO has developed the ICOPE and ICOPE Monitor apps as vehicles to continuously assess and monitor the intrinsic capacity of older adults to guide the delivery of person-centered care (7), and the 2022 WHO work report identified the digital integration of health information as an enabler of ICOPE implementation (8). In addition, Vestjens et al. (9, 10) pointed out that accessible ICT is key to the sustainable spread of proactive integrated care and that integrated care services for frail older adults based on electronic case systems, GP information systems, and chain information systems significantly improve the quality of care for frail older adults, but these services are not cost-effective. The SmartCare project, which targets older adults in 16 European countries, integrates care based on electronic record systems, email, telephone, and fax, and has significantly reduced the number and length of hospital stays for older adults and the cost of care for caregivers (11). Ruikes et al. (12) confirmed that there was no net monetary benefit to the CareWell primary care program for frail older adults based on the eHealth case, health, and wellbeing information portal, and that it had no significant impact on improving older adults' activity functioning, quality of life, mental health, institutionalization, hospitalization, or mortality, and no observed effect on improving caregiver quality of life or caregiving burden. Conflicting findings on the effectiveness of ICT-based integrated care for older adults are apparent, and further systematic evaluation is needed to clarify the effectiveness of the intervention.

The research team conducted a scoping review of the ICT-based integrated care model for older adults. A descriptive analysis of articles with quantitative, qualitative, and mixed-method research designs found positive impacts on quality of life, caregiver burden, and primary care resource utilization among older adults, but the validity assessments were not as robust as hoped. Heterogeneity was high, and 60.5% of the included studies were empirical studies, necessitating systematic evaluation to further validate their effectiveness (13). In addition, existing studies have focused on the effectiveness of preventive integrated care for frail older adults in the community (14), the core components and impact of nurse-led models of integrated care for older adults at home (15), and systematic evaluations of service providers' perceptions of integrated care for frail older adults (16). However, there is a lack of systematic reviews of the effectiveness of integrated care services for older adults based on ICT as a form of practice. Therefore, this review systematically synthesizes the best available evidence related to the implementation of ICT-based integrated care for older adults to evaluate the effectiveness of this intervention for older adults compared to usual care and usual primary care.

## 2 Material and methods

We followed the JBI Evidence Synthesis Manual (17) and PRISMA guidelines for reporting the results of the systematic review.

### 2.1 Search strategy and selection criteria

We conducted a systematic search of nine databases—PubMed, Web of Science Core Collection, Scopus, MEDLINE, EBSCO, EMBASE, CINAHL with full text, ProQuest, and the Cochrane Library—for articles published in English between January 1, 2000, and July 25, 2022. We also manually searched Google Scholar to identify unpublished articles and conducted positive citation tracking and reference list screening for the studies to be included. Common phrases were identified using Medical Subject Headings (MeSH). Selected subject headings included age, information technology, “delivery of health care, integration”, randomized controlled trials as a topic, and non-randomized controlled trials as a topic. An initial search was conducted in the PubMed database by reading relevant text to identify synonyms of the subject terms and using Boolean logic operators to combine the subject terms and free words for the literature search (see Supplementary material S2 for detailed search terms and Supplementary material S3 for details of the search strategy for each database).

The inclusion criteria were developed following the PICOS principles recommended by the Cochrane systematic review as follows: (1) intervention/service targets were older adults aged 60 years and older; (2) ICT-based integrated care interventions used ICT as a platform for care delivery, information sharing, and communication to support the provision of integrated care services, and the study had to be within the context of ICT and integrated care, including any health care setting (e.g., primary health care, hospital, emergency department or health care consortium); (3) routine primary care as the control group; (4) outcome indicators included the primary outcome (quality of life, mobility, depression status, medical resource utilization (readmission, hospitalization, emergency care), mortality) and secondary outcomes (cost-effectiveness, quality of care, primary care resource use); and (5) a randomized controlled trial (RCT) or quasi-experimental study. The exclusion criteria were (1) studies with nonhuman subjects; (2 other study designs (e.g., cohort, qualitative, review, editorial); and (3) literature with incomplete data reporting or where full text was not available. If the meeting abstract and trial details of the study protocol met our inclusion criteria, an email was sent to the corresponding author or trial leader to obtain a detailed report of the study results.

### 2.2 Study screening and selection

The search results were sequentially imported into NoteExpress literature management software for checking. Two reviewers (TYT, MLX) read the article titles and abstracts for initial screening based on the inclusion and exclusion criteria. The full text was then located and downloaded, and two reviewers (TYT, MLX) independently read the full text for rescreening. In case of disagreement, consensus was reached through discussion with a third reviewer (LXH).

### 2.3 Critical appraisal/quality assessment

Two reviewers (TYT, MLX) used a standardized critical appraisal tool developed by JBI to evaluate the quality of the literature for inclusion in four categories: yes, no, unclear, and not applicable. A lead reviewer (TYT) first pre-evaluated three to five papers, and two reviewers (TYT, MLX) negotiated possible scenarios for each evaluation item, refining the evaluation criteria to form a unified standard. Disagreements between reviewers were resolved through discussion with a third reviewer (LXH). The reviewers screened the literature based on a predetermined proportion of included studies that met all criteria with the stipulation that those below 60% would be removed. The final methodological quality of each study and the risk of bias for each aspect was reported in tabular form.

### 2.4 Data extraction

Two reviewers (TYT, MLX) performed data extraction independently using a standardized data extraction form developed by JBI that included the author name, year of publication, country of affiliation, literature title, study design, study purpose, study scenario, study population, sample size, intervention, control measures, assessment time points, primary and secondary outcome indicators, and intervention results. Once the data were extracted, they were checked by the review team. For studies where there was no consensus, a third reviewer (LXH) read the full text and participated in discussion to find a solution. If necessary, the reviewer sent an email to the study authors to clarify the data, request missing data, or perform calculations using the data conversion method.

### 2.5 Statistical analysis

We analyzed the heterogeneity of the studies from both clinical and methodological perspectives. Descriptive/narrative analyses were conducted for the more heterogeneous outcome indicators, and meta-analyses were conducted for the less heterogeneous outcome indicators. Random effects models were used to calculate changes in quality of life, mobility, hospitalization, readmission, emergency department visits, and death before and after the intervention, and continuous outcome variables were combined using mean differences (MDs) or standardized mean differences (SMDs). Heterogeneity between studies was quantified using the I^2^ statistic, with effect sizes and corresponding indicators visualized by forest plots. All meta-analysis methods were performed using RevMan (Review Manager (RevMan) 5.3. Copenhagen: The Nordic Cochrane Center, The Cochrane Collaboration, 2014).

## 3 Results

**Figure 1:**
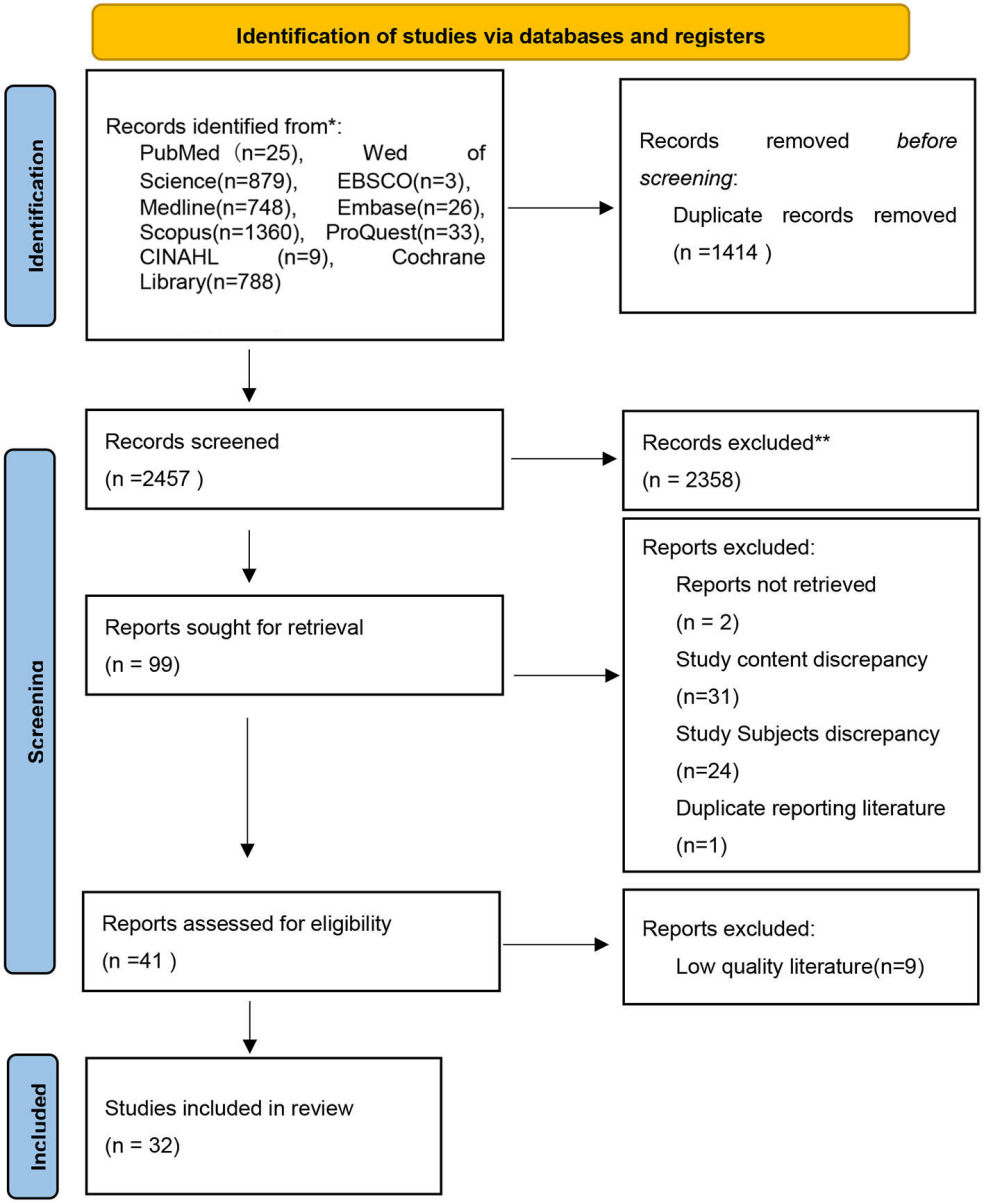
Flow diagram of literature screening.

We retrieved a total of 3,871 studies from nine databases. Of these, 1,414 duplicates were eliminated, and 2,457 articles were initially screened by reading titles and abstracts. We excluded 2,358 articles that did not meet the inclusion criteria, and we further downloaded and read the full text to rescreen 99 articles. We further excluded nine articles that were of low quality, two articles that were not available in full text, 55 articles that did not meet the inclusion criteria, and one duplicate article. Finally, 32 articles were included. The flow chart of the literature screening is detailed in Figure 1.

### 3.1 Literature quality assessment

Nine studies had a predetermined percentage of JBI quality evaluation criteria below 60% and deficiencies in randomization, allocation concealment, blinding, treatment of missed visits and comparability of interventions. These were removed after panel discussion. Of the 15 RCTs, only one study was blinded to the study population (18), seven studies were blinded to the outcome measure (19–25), and nine studies achieved allocation concealment (18, 20, 22–24, 26–29), which still left some risk of bias in their selection, implementation, and measurement. Sixteen of the 17 quasi-experimental studies had a control group (30–45), but only five studies were comparable between groups at baseline, and the results should be discussed in conjunction with the analysis (30, 32, 36, 45, 46). The results of the quality assessment of the literature are detailed in Tables 1, 2.

**Table 1:** Results of quality assessment of RCTs.

**References**	①	②	③	④	⑤	⑥	⑦	⑧	⑨	⑩	⑪	⑫	⑬	**Total**
Uittenbroek et al. (47)	Y	U	Y	N	N	N	Y	Y	Y	Y	Y	Y	Y	9/13
Spoorenberg et al. (19)	Y	U	Y	N	N	Y	Y	Y	Y	Y	N	Y	Y	9/13
Uittenbroek et al. (20)	Y	Y	Y	N	N	Y	Y	Y	Y	Y	Y	Y	Y	11/13
Kim et al. (21)	Y	N	Y	N	N	Y	Y	Y	Y	Y	Y	Y	Y	10/13
Tu et al. (18)	Y	Y	Y	Y	N	N	Y	Y	Y	Y	N	Y	Y	10/13
Dolovich, et al. (26)	Y	Y	N	N	N	N	Y	Y	Y	Y	Y	Y	Y	9/13
Barker et al. (22)	Y	Y	Y	N	N	Y	Y	Y	N	Y	Y	Y	Y	10/13
Di Pollina et al. (48)	Y	N	Y	N	N	N	Y	Y	Y	Y	Y	Y	Y	9/13
Chan et al. (23)	Y	Y	Y	N	N	Y	n/a	Y	Y	Y	Y	Y	Y	10/13
Boult et al. (24)	Y	Y	N	N	N	Y	Y	N	Y	Y	Y	Y	Y	9/13
Boult et al. (27)	Y	Y	N	N	N	U	Y	N	Y	Y	Y	Y	Y	8/13
Boyd et al. (28)	Y	Y	N	N	N	U	Y	N	Y	Y	Y	Y	Y	8/13
Boorsma et al. (25)	Y	N	N	N	N	Y	Y	Y	Y	Y	Y	Y	Y	9/13
Liang et al. (49)	Y	N	Y	N	N	N	Y	Y	Y	Y	N	Y	Y	8/13
Mary et al. (29)	Y	Y	Y	N	N	N	Y	Y	N	Y	Y	Y	Y	9/13
	15/15	9/15	10/15	1/15	0/20	7/20	14/15	12/15	13/15	15/15	12/15	15/15	15/15	

Y, Yes; N, No; U, Unclear; n/a, Not Applicable.

① Was true randomization used for assignment of participants to treatment groups? ② Was allocation to treatment groups concealed? ③ Were treatment groups similar at the baseline? ④ Were participants blind to treatment assignment? ⑤ Were those delivering treatment blind to treatment assignment? ⑥ Were outcomes assessors blind to treatment assignment? ⑦ Were treatment groups treated identically other than the intervention of interest? ⑧ Was follow up complete and if not, were differences between groups in terms of their follow up adequately described and analyzed? ⑨ Were participants analyzed in the groups to which they were randomized? ⑩ Were outcomes measured in the same way for treatment groups? 11 Were outcomes measured in a reliable way? 12 Was appropriate statistical analysis used? 13 Was the trial design appropriate, and any deviations from the standard RCT design (individual randomization, parallel groups) accounted for in the conduct and analysis of the trial?

**Table 2:** Results of quality assessment of quasi-experimental studies.

**References**	①	②	③	④	⑤	⑥	⑦	⑧	⑨	**Total**
Colomina et al. (30)	Y	Y	Y	Y	Y	Y	Y	N	Y	8/9
Piera-Jimenez et al. (31)	Y	N	Y	Y	Y	N	Y	Y	Y	7/9
Mateo-Abad et al. (32)	Y	Y	Y	Y	Y	Y	Y	Y	Y	9/9
Mateo-Abad et al.^①^(33)	Y	N	Y	Y	Y	Y	Y	Y	Y	8/9
Vestjens et al. (34)	Y	N	Y	Y	Y	Y	Y	Y	Y	8/9
Ruikes et al. (36)	Y	Y	Y	Y	Y	Y	Y	N	Y	8/9
Ruikes et al. (35)	Y	N	Y	Y	Y	Y	Y	N	Y	7/9
Ruikes et al. (37)	Y	N	Y	Y	Y	Y	Y	N	Y	7/9
Looman et al.^②^ (38)	Y	N	Y	Y	Y	Y	Y	Y	Y	8/9
Looman et al.^③^ (39)	Y	N	Y	Y	Y	Y	Y	Y	Y	8/9
Looman et al. (40)	Y	N	Y	Y	Y	U	Y	Y	Y	7/9
Bakker et al. (46)	Y	Y	n/a	N	Y	Y	Y	Y	Y	7/9
Sylvia et al. (41)	Y	N	U	Y	Y	N	Y	Y	Y	6/9
Boyd et al. (42)	Y	N	Y	Y	Y	N	Y	Y	Y	7/9
Hébert et al. (43)	Y	N	U	Y	Y	Y	Y	Y	Y	7/9
Hullick et al. (44)	Y	U	Y	Y	N	N	Y	Y	Y	6/9
Tourigny et al. (45)	Y	Y	U	Y	Y	Y	Y	N	Y	7/9
	17/17	5/17	13/17	16/17	17/17	13/17	17/17	13/17	17/17	

① Is it clear in the study what the ‘cause' and what the ‘effect' are (i.e., there is no confusion about which variable comes first)? ② Were the participants included in any comparisons similar? ③ Were the participants included in any comparisons receiving similar treatment/care, other than the exposure or intervention of interest? ④ Was there a control group? ⑤ Were there multiple measurements of the outcome both pre and post the intervention/exposure? ⑥ Was follow up complete and if not, were differences between groups in terms of their follow up adequately described and analyzed? Ɇ Were the outcomes of participants included in any comparisons measured in the same way? ⑧ Were outcomes measured in a reliable way? ⑨ Was appropriate statistical analysis used.

### 3.2 Characteristics of included studies

We reviewed 14 quasi-experimental studies (30–34, 38–43, 45, 46, 48), six cluster-randomized control trials (cRCTs) (18, 21, 24, 25, 27, 28), four cluster nonrandomized control trials (35–37, 44), and eight RCTs (19, 20, 22, 23, 26, 29, 47, 49). Sample sizes ranged from 69 to 18,837 (14,289 in the control group and 15,911 in the intervention group). The study population was mainly frail older adults (40.6%) and chronically ill older adults (37.5%). Seven studies focused specifically on older adults at high risk of health care resource use and functional decline (24, 27, 28, 32, 33, 43, 49). Study settings included GP practice (43.8%), home (21.9%), hospital (18.8%), hospital to home transition (12.5%), community (9.4%), and nursing facility (9.4%).

Thirty-two studies provided integrated care based on ICT through the formation of multidisciplinary teams, with interventions ranging from 3 months to 4 years. Recruited team members included practice nurses, clinicians, GPs, primary care nurses, pharmacists, rehabilitation practitioners, psychologists, dieticians, and social workers (e.g., volunteers, social workers). In 14 studies, dedicated case manager positions were used to oversee, organize, and coordinate the implementation of interventions to provide case management services for older adults (19, 20, 30, 31, 34–40, 43, 45, 47). The information and communication technologies used were mainly telephone (62.5%), clinical information systems (40.6%), electronic health records (40.6%), electronic medical records (15.6%), personal health records (9.4%), and online health education materials for patients and carers.

The core elements of Integrated care can be grouped into seven dimensions: single entry point, comprehensive geriatric assessment, individualized care planning, multidisciplinary case conferences, coordination of care, case management, and patient empowerment. Researchers often identify primary care practices and health professionals as a single entry point to increase coordination and continuity of care. Through a multidimensional assessment of the functional health and care needs of older adults and their caregivers, individualized care plans are developed based on participants' preferences, health issues, and priorities. Furthermore, regular interdisciplinary case conferences are organized with members of the multidisciplinary team to develop and adapt care plans and coordinate care delivery with health and social agencies and medical and social workers, during which patients are empowered and encouraged to participate in the process of integrating care practices. The general characteristics of the included studies are detailed in Supplementary material S1.

### 3.3 Primary outcomes

#### 3.3.1 Quality of life

**Figure 2:**
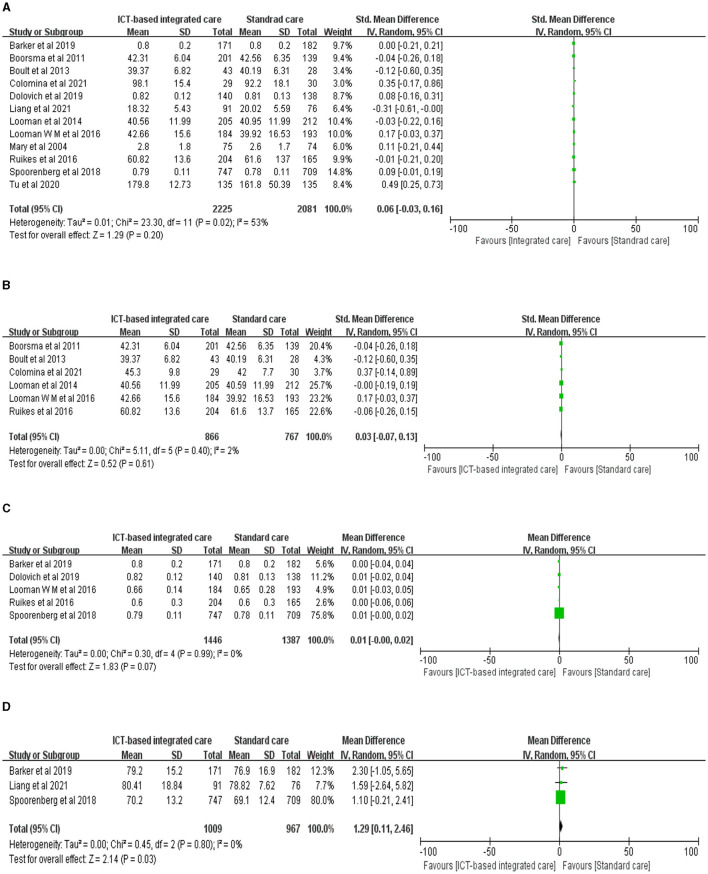
Forest plot of the intervention effect of ICT-based integrated care on quality of life for older adults. **(A)** Quality of life. **(B)** SF. **(C)** EQ-5D. **(D)** EQ-VAS.

Overall, ICT-based implementation of integrated care services had no significant impact on improving the quality of life of older adults compared to usual care and primary care [Figure 2A, *n* = 12 studies, SMD 0.06 (CI −0.03 to 0.16), moderate heterogeneity]. However, there was some heterogeneity in the integration results, and further descriptive analysis revealed that five studies significantly improved the quality of life of older adults. Tu et al. (18) found a more significant intervention effect, based on telephone coordination that provided two-way referral and 6-month post-discharge support for older adults with diabetes during the hospital–home transition. Subgroup analysis according to the measurement tool found significant improvements in the overall perceived health status of older adults [Figure 2D, *n* = 3 studies, MD 1.29 (CI 0.11 to 2.46), no heterogeneity], but no significant changes in the five dimensions of self-rated health: mobility, self-care, daily activities, pain/discomfort, and anxiety/depression [Figure 2C, *n* = 5 studies, MD 0.01 (CI −0.00 to 0.02), no heterogeneity]. The study by Spoorenberg et al. (19), which had the largest weighting and longest intervention (12 months), recruited GPs, geriatric care doctors, and case managers (social workers, district nurses) in GP clinics to develop and implement individualized care and support plans based on a clinical information system, an electronic record system for older adults, and an assessment of the complexity of care needs and vulnerability of older adults, effectively counteracting the physical, cognitive, and social decline associated with aging. In addition, the results of six studies using the Short Form Health Scale to measure quality of life did not find significant changes [Figure 2B, *n* = 6 studies, MD 0.03 (CI −0.07 to 0.13), no heterogeneity], [*n* = 6 studies, MD 0.03 (CI −0.07 to 0.13), no heterogeneity], but Looman et al. (39, 40) showed that integrating care improved the attachment dimension of quality of life and had a positive impact on love and friendships.

#### 3.3.2 Mobility

**Figure 3:**
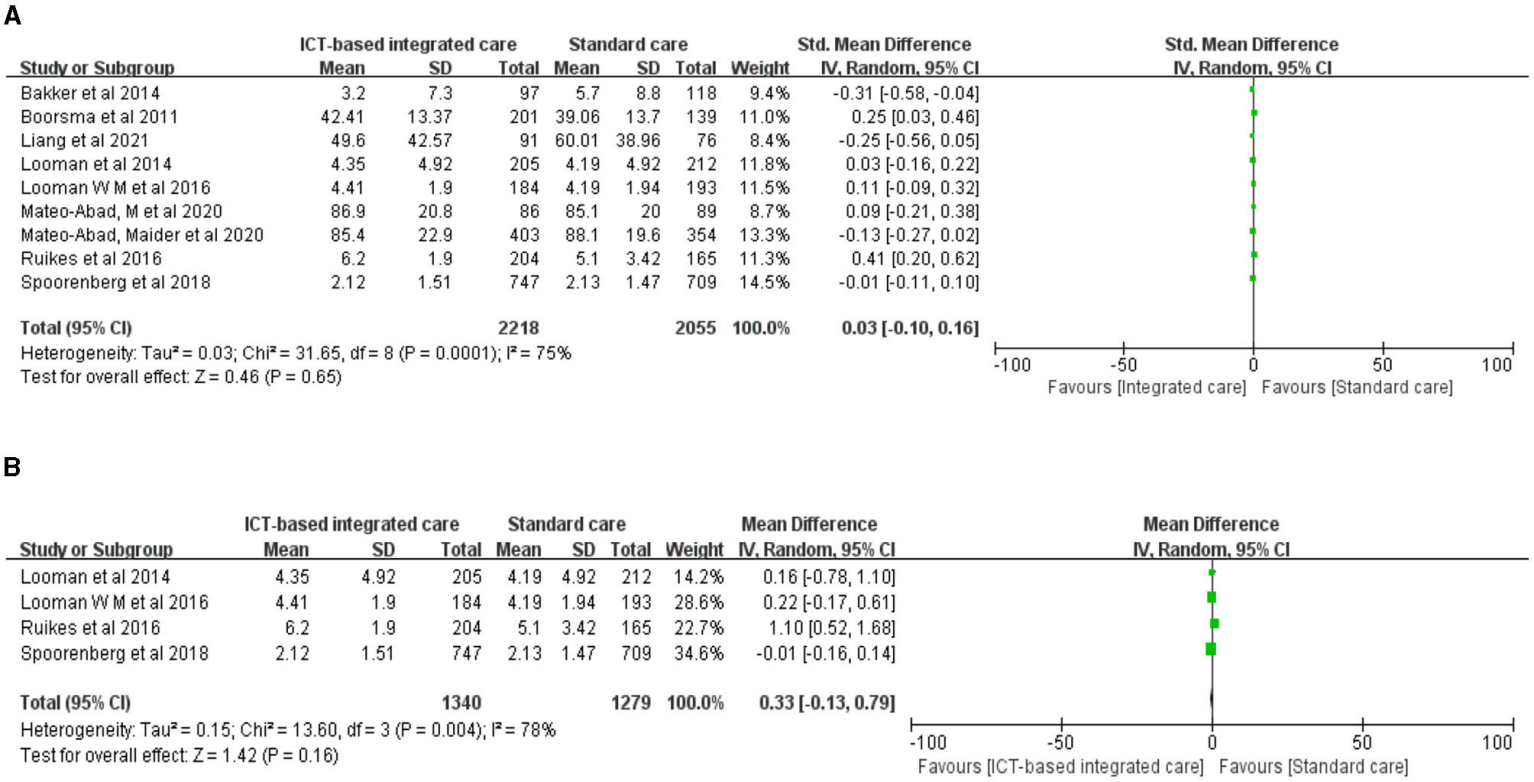
Forest plot of the intervention effect of ICT-based integrated care on mobility for older adults. **(A)** Mobility. **(B)** Katz-15.

We found no evidence that ICT-based integrated care improved mobility in older adults (Figure 3A, *n* = 9 studies, SMD 0.03 (CI −0.10 to 0.16), high heterogeneity). Bakker et al. (46) had a shorter intervention length (3 months), but their CareWell integrated care program for frail older adults based on a clinical health care information system significantly improved the mobility of older adults after the intervention; the system included frailty screening and clinical judgment, patient medical information and medication assessment, development and updating of CareWell plans, proxy medical records, comprehensive geriatric assessment, multidisciplinary meetings, volunteer-assisted cognitive and physical activity, and health care worker education and job coaching. The integration of four studies that used the Katz-15 index to assess daily living skills also found no significant effects (Figure 3B, *n* = 4 studies, MD 0.33 (CI −0.13 to 0.79), high heterogeneity), but there was considerable heterogeneity in the results, and the small number of included studies did not allow for further subgroup analysis.

#### 3.3.3 Depression status

**Figure 4:**
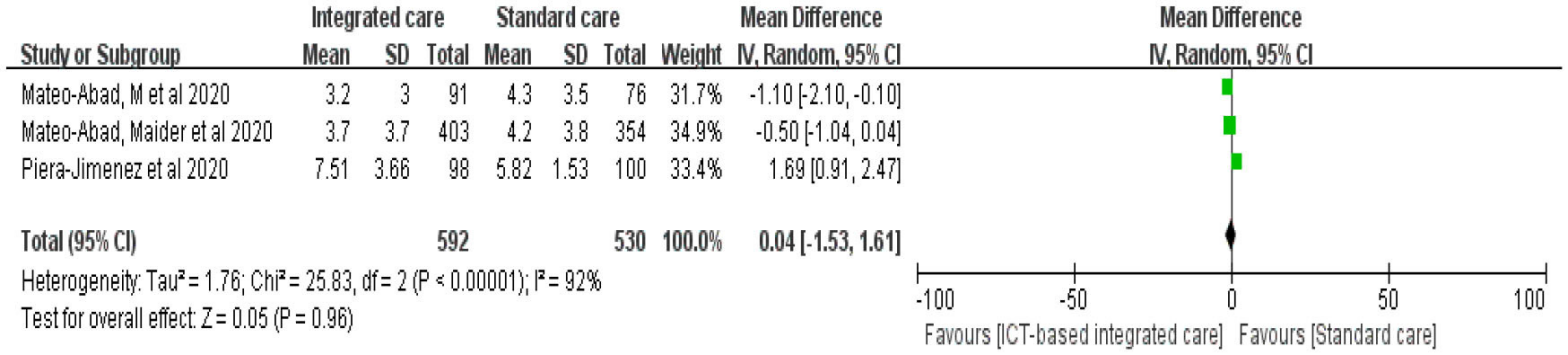
Forest plot of the intervention effect of ICT-based integrated care on depression status for older adults.

Three studies evaluated the effect of ICT-based integrated care using the Geriatric Depression Scale, but the results were heterogeneous and did not show a significant effect on improving depression in older adults (Figure 4, *n* = 3 studies, MD 0.04 (CI−1.53 to 1.61), high heterogeneity). The only study that showed evidence of an effect had the smallest weight and sample size: Mateo-Abad et al. (32, 33) developed a patient empowerment and home care (KronikOn empowerment program) program based on multiple chronic conditions in older adults, which may have improved depression in older adults to some extent.

#### 3.3.4 Medical resource utilization

**Figure 5:**
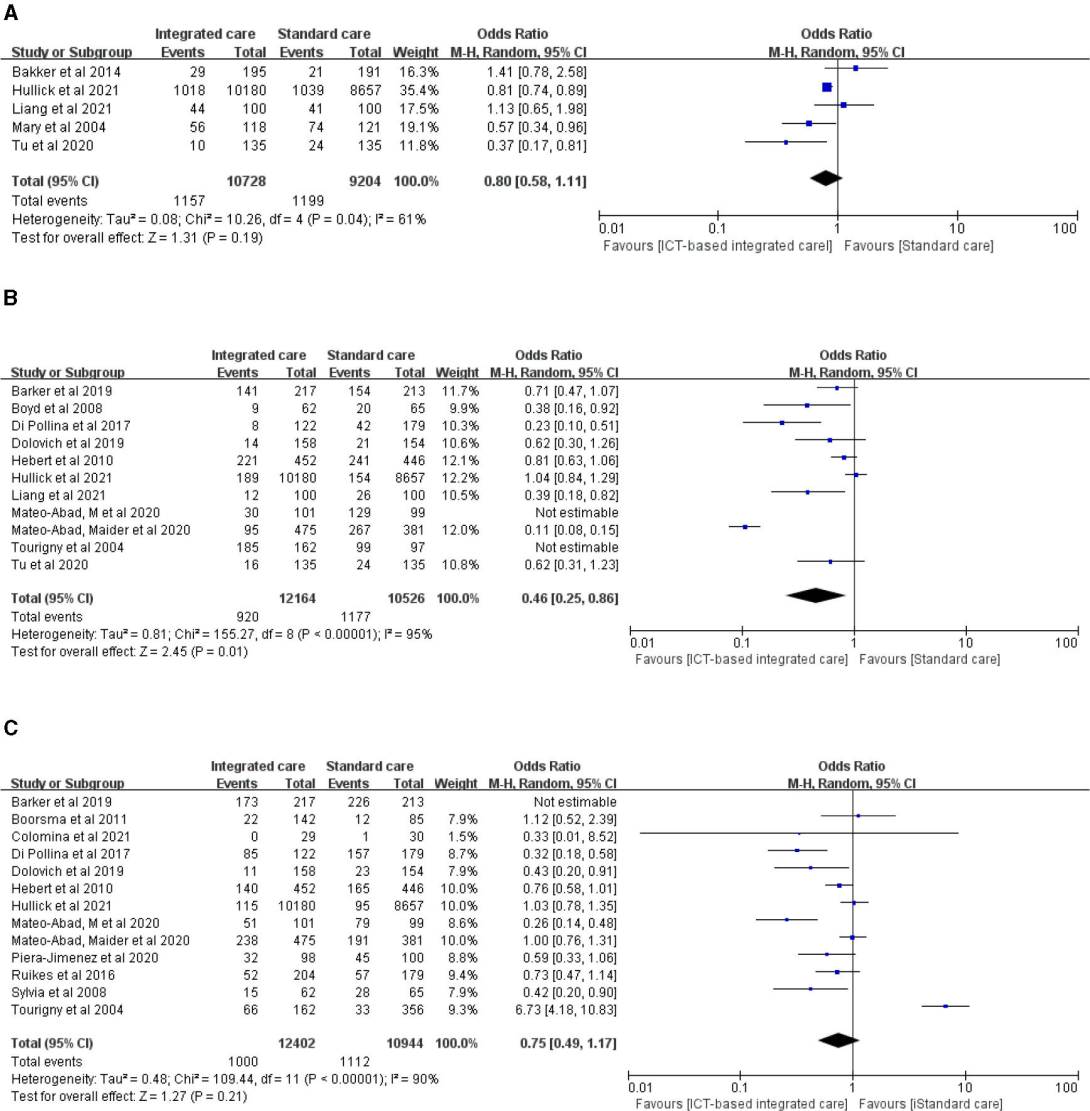
Forest plot of the intervention effect of ICT-based integrated care on medical resource utilization for older adults. **(A)** Readmission. **(B)** Emergency department visit. **(C)** Hospitalization.

We evaluated health care resource use in terms of readmissions, emergency department visits, and hospitalizations. Five studies evaluated readmissions in older adults before and after interventions, but the pooled results showed no significant reduction in readmissions (Figure 5A, *n* = 5 studies, OR 0.80 (CI 0.58 to 1.11), moderate heterogeneity). Mary, Tu et al. (18, 29) focused on integrated coordinated care interventions for older adults with heart failure and diabetes during the transition from hospital to home. These interventions significantly reduced the total number of readmissions. The length of intervention in two studies was 3 months and 6 months, respectively. Thirteen studies evaluated the number of hospital admissions and found no strong evidence to indicate that hospital admissions were reduced (Figure 5C, *n* = 13 studies, OR 0.75 (CI 0.49 to 1.17), high heterogeneity), but there was high heterogeneity in the pooled results. Further descriptive analysis revealed that six of these studies significantly reduced the number of unplanned visits and hospital admissions for older adults (26, 30, 32, 43, 44, 48).

Additionally, we found that ICT-based integrated care services significantly reduced the number of emergency department visits for older adults (Figure 5B, *n* = 11 studies, OR 0.46 (CI 0.25 to 0.86), high heterogeneity). However, two studies with emergency visits larger than the sample size were not included in the meta-analysis. Mateo-Abad et al. (32, 33) implemented a 12-month CareWell integrated care service in Spain for older adults with multiple chronic conditions and complex risks through the identification of frail older patients, comprehensive baseline assessment, development of individualized plans, programmed follow-up, home support patient stabilization, integrated care during hospitalization, and a coordinated discharge service pathway, significantly reducing emergency department visits. Tourigny et al. (45) provided interdepartmental coordination at the strategic, tactical, and clinical levels to provide integrated services for frail older adults over a 3-year period based on a single entry point, a single patient assessment tool, case management, development of individualized service plans, and computerized clinical icons. These authors showed that the intervention group had a lower rate of return to emergency care within 10 days of the initial visit, but there was no significant effect on the overall number of emergency department visits.

#### 3.3.5 Mortality

**Figure 6:**
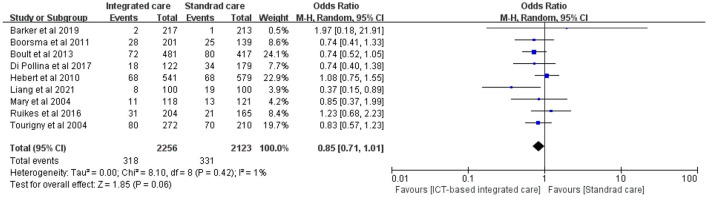
Forest plot of the intervention effect of ICT-based integrated care on mortality for older adults.

Nine studies evaluated the effect of ICT-based integrated care on mortality in older adults, and mortality in the intervention group was smaller than that in the control group, but the difference was not statistically significant (Figure 6, *n* = 9 studies, OR 0.85 (CI 0.71 to 1.01), low heterogeneity). The most effective study was by Liang et al. (49), whose wireless transmission device-based tele-home care program for the remote monitoring of physiological indicators, medication management, emergency calls, early warning of abnormal conditions, nurse assessment, and coordination of care for older adults with multiple chronic conditions significantly reduced mortality.

### 3.4 Secondary outcomes

Eight studies evaluated the cost-effectiveness of ICT-based integrated care services, and four studies (29–31, 41) confirmed that they could reduce insurance expenditures, lower health care costs, and potentially improve clinical and economic outcomes in high-risk older adults. However, the remaining four studies (34, 36, 38, 47) showed higher average total costs and smaller improvements in health-related outcomes in the intervention group and no net monetary benefits of ICT-based integrated care. In terms of quality of care evaluation, seven studies (20, 21, 24, 25, 28, 42, 48) showed significant improvements in the overall quality of care for older adults and suggested that ICT-based integrated care improved goal setting, coordination, and decision support for health care for older adults. In addition, seven studies (18, 19, 21–23, 43, 45) showed that ICT-based integrated care offset declines in physical, cognitive, communication, and social functioning associated with aging and frailty, with significant increases in satisfaction, empowerment, and the proportion of needs met. Three studies (26, 32, 33) showed significant increases in the use of primary care services by older adults.

## 4 Discussion

ICT-based integrated care services can, to some extent, improve the perceived health status of older adults, reduce the number of emergency department visits, and improve the overall quality of care. However, their effects on quality of life, mobility, depression, readmission and hospitalization, mortality, and economic costs are not significant. In contrast, 3- or 6-month short-term ICT-based integrated care services have an impact on improving the quality of life, number of readmissions, and mobility of older adults in the transition from hospital to home.

Five studies showed that ICT-based integrated care can significantly improve the health-related quality of life of older adults, a finding confirmed by Rajan et al. (50) in their evaluation of the effectiveness of integrated care for Parkinson's patients. This may be related to the fact that the effective interface between medical and social resources in integrated care can significantly improve the quality of care. ICTs provide a platform to facilitate communication and coordination among institutional, organizational, and multidisciplinary team members, and well-organized coordination activities are positively associated with patients' health-related quality of life (51, 52). However, ICT-based integrated care had no significant impact on the five dimensions of self-rated health status of older people: mobility, self-care, daily activities, pain/discomfort, and anxiety/depression. This may be related to the fact that the EQ-5D primarily evaluates physical functioning, whereas integrated care targets psychological functioning and social wellbeing (53). Moreover, the intervention in this study focused on frail, chronically ill older adults, whose intrinsic capacity declines with age and who are at increased risk of functional decline and poor health outcomes. Ma et al. (54) confirmed that declining intrinsic capacity is associated with frailty and incapacity and is accompanied by a decline in physical, mental, and overall health, and that higher intrinsic capacity is associated with a better quality of life. However, seven studies showed that ICT-based integrated care can offset the decline in physical, cognitive, communication, and social functioning associated with aging and frailty. Researchers could develop ICT-based integrated care programs based on an assessment of intrinsic capacity by taking into account older people's environment to achieve functional performance.

The meta-analysis results showed that ICT-based integrated care did not have a significant impact on improving the mobility and depression of older adults, although one short-term intervention study showed a positive effect. This may be because older adults' mobility declines as the duration of the intervention increases (55), which influences the effect of the intervention. Rietkerk et al. (56) empirically showed that goals related to mobility and pain were the least likely to be achieved in an integrated care goal plan, which affected the improvement of mobility in older adults. Cognitive decline, depression, and limited mobility are severe threats to the intrinsic abilities of older adults (54). González-Bautista et al. (57) found that 89.3% of older adults suffered from one or more disorders related to intrinsic decline, including cognitive decline in 52.2% of cases, depressive symptoms in 39%, and mobility problems in 20.2%. Multiple disorders are associated with higher levels of functional limitation and depression (58). Yu et al. (59) showed that cognitive decline and mobility problems in older adults predicted emergency department visits and that mobility problems predicted poorer quality of life. In addition, depression in older adults can lead to significant morbidity, which can affect physical and mental health, overall health outcomes, daily functioning and quality of life (60). However, only five studies (25, 38–40, 48) recruited psychologists to form multidisciplinary teams and did not specifically provide care services for psychological problems in older adults, instead recommending comprehensive initial assessment using standardized rating scales, follow-up monitoring, the provision of care manager-led psychological support techniques (61, 62), and the development of goal plans to improve physical and mental health and mobility-related health problems.

ICT-based integrated care had no significant effect on reducing readmission, hospitalization, or mortality in older people. However, the number of positive events was smaller in all intervention groups than in the control group, similar to the findings of Jepma et al. (63). Two studies showed significant reductions in patient readmissions, with ICT-based integrated care scenarios in the transition from hospital to home, but with shorter study interventions, indicating that they could reduce the number of readmissions in the short term for older patients discharged from the hospital. Five studies confirmed the positive effect of ICT-based integrated care on reducing the number of unplanned visits and hospital admissions. This suggests that ICT-based integrated care may reduce the use of health care resources by older adults in some cases and may be associated with improved health outcomes for older adults. Sood et al. (64) also demonstrated that vertically integrated hospital and rehabilitation facility care can reduce the length of stay and maintain or improve health outcomes. However, the results of a longitudinal matched study showed that integrating care was unlikely to lead to improved survival or reduced emergency admissions (65). Morciano et al. (66) evaluated two large nationally initiated service integration programs in England (Pioneer and Vanguard) and found that they mitigated but did not prevent an increase in long-term emergency admissions.

The meta-analysis results of this study showed a significant decrease in the number of emergency department visits and an increase in the frequency of use of primary health care services among older adults in the intervention group, consistent with the findings of Stephenson et al. (67). This result may be related to the gradual improvement or stabilization of the functional status in older adults, the increasing demand for long-term care, the gradual shift in health care decisions toward primary health care, and the use of ICT, which can provide continuous telehealth monitoring and guidance for older adults. This may include abnormal value warning settings that can assist health care workers in dealing with emergencies remotely and avoiding emergency admissions for older patients.

The economic cost-effectiveness of ICT-based integrated care is unclear. Four studies confirmed its ability to reduce insurance and medical expenditures for older people, which may be associated with a reduced risk of hospitalization and an increased frequency of use of primary care services. Primary care institutions are the core organizations in vertical integration, and primary health care provides a cost-effective way to achieve coverage (68). However, four studies showed higher costs of ICT-based integrated care interventions and correspondingly less change in health outcomes. This may be due to the short duration of the interventions included in the studies, which were only 6–12 months long, and the fact that the interventions were mostly targeted at frail or older adults with multiple chronic illnesses who may not be able to respond to the interventions in the short term. Corresponding improvements in health outcomes were not observed. Furthermore, a proactive approach to implementing an intervention may increase older adults' awareness of care needs and their early use of services and informal care, resulting in higher costs for the intervention group and higher costs per quality-adjusted life year (69). Recruiting multidisciplinary team members for intervention delivery also increases the human cost expenditure. However, Batlle et al. (70) noted that linking primary, hospital, and social care professionals to implement an integrated care model with patient-centered mobile health support could reduce unintended contact with the health system and health costs. The commitment of health and social care professionals to new models of care can enhance the effectiveness of interventions. It is therefore necessary to develop highly integrated and responsible care organizations, seek public funding if necessary, and promote the implementation of integrated care in the form of government purchases of services.

### 4.1 Limitations and outlook of this study

The quality of the literature for the studies included in this review needs to be improved, and blinding and allocation concealment practices need to be improved. It is recommended that researchers ensure that studies are conducted with comparable baseline intervention and control groups to reduce the risk of selection and implementation bias. In addition, the overall heterogeneity of the studies we included was too large to consider the impact of ICT-based integrated care on health outcomes and economic costs for older adults as a whole, consistent with the findings of a systematic evaluation of integrated care by Rocks et al. (71). Follow-up researchers should integrate local health and social welfare provision systems, recruit multidisciplinary team members based on the needs of the study population, and develop an intelligent integrated care model guided by preferences and priorities.

## 5 Conclusion

ICT-based integrated care can significantly improve perceived health status, emergency visits, and quality of care for older adults, but the effects of interventions on quality of life, mobility, depression, readmission and hospitalization, mortality, and economic costs are unclear. However, the implementation of ICT-based integrated care for older adults in the hospital-to-home transition produced some short-term effects. Subsequent researchers can focus on the scenario of hospital-to-home transition and conduct systematic reviews or empirical studies on ICT-based integrated care services to explore their short- or long-term effects. Furthermore, the available evidence is heterogeneous, the quality of the literature should be improved, and subsequent studies should strictly follow the PRISMA guidelines for reporting results to improve the level of evidence.

## Data availability statement

The original contributions presented in the study are included in the article/Supplementary material, further inquiries can be directed to the corresponding author.

## Author contributions

YTT: Conceptualization, Data curation, Methodology, Visualization, Writing—original draft, Writing—review & editing. SSW: Data curation, Methodology, Resources, Visualization, Writing—review & editing. YZ: Conceptualization, Funding acquisition, Methodology, Writing—review & editing. LXM: Data curation, Formal analysis, Resources, Writing—review & editing. XHL: Data curation, Formal analysis, Resources, Writing—review & editing.
