# Supplementary material for: Effectiveness of information and communication technology-based integrated care for older adults: a systematic review and meta-analysis
Source: Front Public Health. 2024 Jan 5;11:1276574. doi: 10.3389/fpubh.2023.1276574 (PMC10797014; doi:10.3389/fpubh.2023.1276574)
Supplement: Supplementary file 1 [file Table_1.DOCX]

# Supplementary materials S1——Description of included studies

| **Author / Year / Country** | **Study Design** | **Study Setting** | **Study Subject** | **Nc** | **Ni** | **Multidisciplinary Team Member** | **ICT** | **Intervention Duration/ Content** | **Outcome Measures** | **Study Results** |
| --- | --- | --- | --- | --- | --- | --- | --- | --- | --- | --- |
| Colomina, Jordi et al.  (29) (2021)  Spain | Prospective, pragmatic, parallel controlled trials | Hospital to home transition | Patients after primary total hip or knee arthroplasty | 30 | 39 | Family doctors, hospital surgical teams and social workers, case managers | Smart Adaptive Case Management (SACM) system, Mobile Self Management for Patient Empowerment (MSMPE), Fitbit Flex 2 digital activity tracker, digital pulse oximeter, digital gauge and digital blood pressure monitor | 3 months  1. Initial assessment of patient health status for key chronic conditions and social needs indicators; 2. MSMPE-based generation of health status reports, virtual coaching with automated feedback and comprehensive communication with the care team; 3. Automated tracking of patient activity and integration into MSMPE; 4. Care team members use the SACM platform to share patient profiles, coordinate professional service providers and establish communication channels; 5. Case managers oversee the entire care process and act as the main point of contact for patients. | Physiological and Mental Health (SF-12)  Health Care Resource Use  Cost-effectiveness (SF-12) | Significant improvement in patients' quality of life.  Fewer unplanned visits and hospital admissions.  The integrated care programme saved €109.88-126.99 per patient and was cost effective. |
| Hongsoo Kim et al  (20) (2021)  Korea | Stepped wedge cluster randomized trial | Nursing home | Frail elderly | 482 | 431 | Nursing home manager, nurse practitioner, social worker, physiotherapist, occupational therapist and nutritionist, SPEC coordinator (1 nurse + 1 social worker) | SPEC Information System, KaKao Talk APP | 6 months  1.Comprehensive geriatric assessment with needs/risk analysis (interRAI LTCF tool assessment to obtain a customized list of triggering needs/risks for each older adults); 2.Development of an individualized needs-based care plan that is validated after discussion with older adults and/or family members; 3.Multidisciplinary case conferences (team members exchange ideas and opinions on care issues, develop solutions and collaborative action); 4.Coordination of care with family members, external health professionals and agencies; 5. ICT-based sharing of health assessment data, monitoring of intervention progress, and provision of information support. | Quality of care: a composite quality indicator (QI) of the interRAI assessment system | Significant improvement in the overall quality of care for older people.  Effectively prevented deterioration in late ADL loss, cognitive ability, communication skills, new or persistent delirium and behavioural problems. |
| Liang, H Y et al.  (48) (2021)  Taiwan, China | RCT | Hospital to home transition | Older adults with multiple chronic conditions and high risk of readmission | 100 | 100 | 8 senior nurses, doctors and technical engineers | Wireless transmission devices (one-touch smartphone, blood pressure monitor, medication dispenser and emergency call button), telephone | 6 months  1.Wireless transmission-based devices for remote physiological indicator monitoring, medication management, and emergency calls, automatically transmitted to a 24-hour call center and medical records system; 2. Patients communicate with a nurse-led 24-hour call center, check vital signs, and set twice-daily medication reminders via a one-touch smartphone; 3. Individualized alert sets and thresholds are developed for each patient and checked twice daily biological parameters; 4. Initial assessment of symptoms by nurses when abnormal parameters trigger an alert to determine whether to treat the alert as an emergency and coordinate its transfer to emergency services or specialist services; 5. Nurses provide health education, nutritional and medication advice, medication reminders, appointment scheduling with doctors and urgent medical care to patients and carers; 6. Home visits by nurses on discharge day, at 3 months and at 6 months, based on patients' needs for additional visits. | Primary outcome: emergency department visits, readmission and mortality  Secondary outcome: patient medication adherence, activities of daily living(ADL), health status and quality of life | The tele-home care programme significantly reduced mortality and ED visits, with no significant impact on readmission.  Patients' quality of life improved significantly. |
| Hullick, C J et al.(43)  (2021)  Australia | Stepped wedge cluster non-randomized trial | Nursing homes, hospital | Older adults | 8657 | 10180 | Nursing home workers, GPs, A&E nurses, specialist geriatric nurses | Telephone | 12 months  1. 24-hour nurse-led telephone consultation service for RACF staff; 2. Evidence-based algorithms to address common acute symptoms and problems encountered by older people; 3. ED care goals for older adults identified by telephone to clarify reasons for transfer; 4. Specialist geriatric nurses to guide older adults to active case management after admission to the ED; 5. Education on communication skills for RACF staff; 6. A RACF is assigned a home ED; 7. Relationships and collaboration between RACFs, GPs, ambulances, local hospitals and EDs are supported by the community, with quarterly meetings to identify barriers and facilitators to care and regular management and operational meetings with providers and managers; 8. Ongoing change management and coordination of key ACE stakeholders. | Primary outcome: 30-day readmission  Secondary outcome: 7-day emergency department visit, Australian Taxonomy Scale and intensive care admission | ACE programmes can reduce emergency department visits and hospital admissions for older adults with complex health care needs living in RACFs. |
| Piera-Jimenez, J et al(30)  (2020)  Spain | Observational prospective cohort study | Home | Chronically ill older adults | 100 | 98 | Doctors, nurses, social workers, family workers, volunteers and third party groups, case managers | Health Insight Solutions Home Care Platform , Telephone | 8 months  1. Care pathways: (i) integrated short-term home support after an acute episode; (ii) integrated long-term home support. 2. Care processes: ongoing assessment of older people's needs; development and sharing of care plans; single point of entry (case manager); regular visits by professionals or telephone contact with care recipients; ongoing follow-up of older adults' health status based on care plans; deterioration in health status and accidents, automatic on-call team alerts (text messages); third sector care providers share clinical information and provide voluntary companionship support for patients at risk of social exclusion. | Health status(BI), Instrumental Activities of Daily Living Scale and Geriatric Depression Scale  Cost-effectiveness (BI) | The BeyondSilos integrated home care programmer is cost-effective, but it has no meaningful impact on improving activities of daily living, or depression. |
| Tu, Q et al.(17)  (2020)  China | Single blind cluster randomised trial | Hospital to home transition | Geriatric diabetics | 135 | 135 | Medical specialists, GPs, hospital discharge nurses, community nurses | Telephone | 6 months  1. Two-way referral (discharge nurse coordinates and provides post-discharge support for 6 months, community nurse coordinates referral to specialist clinic for medication adjustment); 2. Personalized discharge education in hospital by charge nurse, individualized medication plan by medical specialist prior to discharge, support from GP and community nurse for 6 months after discharge; 3. Learning to monitor, record and report on own health status), home (taking action to achieve self-care goals; monitoring and recording changes in health status in the Patient Edition of the Intervention Diary; regular visits to community health centers or telephone support to communicate self-care processes and outcomes with GPs and community nurses). | Primary outcome: systolic blood pressure  Secondary outcome: health knowledge related to hypertension and diabetes; adherence to medication and healthy lifestyle; glycated haemoglobin (HbA1c); quality of life; incidence of adverse events and complications | Nurse-coordinated transitional care interventions improved hypertension control, HbA1c, knowledge of hypertension and diabetes, treatment adherence, quality of life, readmissions and emergency department visits in older patients with diabetes, but there were no significant differences in adverse events. |
| Mateo-Abad, M et al(31)  (2020)  Spain | Quasi-experimental study | Hospitals and home/health centers | Older adults with multiple chronic conditions and complex risks | 99 | 101 | GPs, social workers, specialists, care managers, primary care nurses (PC) | Electronic health records (EHR), e-prescriptions, e-health call center, personal health records, telephone | 12 months  1. Integrated care pathways: identification of frail elderly patients, comprehensive baseline assessment, development of individualized plans, programmed follow-up (within 24-48 hours of discharge, monthly telephone consultations by PC nurses), home support for patient stabilization, integrated care during hospitalization and coordinated discharge; 2. Care coordination and communication between healthcare providers: setting up care manager roles, ICT-based system for centralized storage of data and sharing of health plans; 3. Patient empowerment and home care: (KronikOn empowerment programmer): four 20-30min basic training sessions in health centers/home, development of optimal self-care plans based on the patient's condition, online access to health education materials. | Number of health care, hospital and emergency center visits; chronic disease diagnoses, health-related parameters, Geriatric Depression Scale, BI | The CareWell integrated care model has transformed health resource use, strengthened the key role of primary care and reduced the number of emergency and hospital admissions.  Satisfaction with the model of care is high. |
| Mateo-Abad, Maider et al.(32)  (2020)  Spain, Croatia, Poland, Italy, UK | Quasi-experimental study | Ibid | Ibid | 381 | 475 | Ibid | E-prescribing, clinician and patient messaging platforms, electronic health records, consultations, call centers, virtual meetings, personal health folders, nurse information systems, education platforms, collaboration platforms, telemonitoring and multichannel centers | 8-12 months  Ibid | Ibid | The CareWell integrated care intervention improved care coordination, patient empowerment and family support, reduced emergency department visits, reduced length of stay and increased use of primary care services (GP and primary care nurse visits were higher than in the control group.) |
| Dolovich, L et al.(25)  (2019)  Canada | Unblinded, pragmatic randomized controlled trial | Home | Older adults | 154 | 158 | Family doctors, medical residents, nurses, pharmacists, various allied health professionals, volunteers (1 person with volunteer experience and 1 university student) | Health TAPESTRY application (TAP-App), electronic medical record (EMR), personal health record (PHR), telephone, e-mail | 6 months  1. Volunteers conduct home visits with participants to discuss older adults' health and life goals, unidentified health and social needs; 2. Primary care team uses TAP-App to collect assessment data, create TAP reports (older adult goals, key messages and/or specific survey scores, volunteer observations); 3. Volunteer coordinator reviews TAP reports and uses TAP-App to securely sent to the TAP-Huddle at the client's clinic; 4. Regular interdisciplinary meetings (TAP-Huddle) to review reports and develop and implement individualized care plans; 5. Community engagement and linkages to assist clients in achieving their goals and addressing health risks and needs. | Primary outcome: goal attainment  Secondary outcome: self-efficacy, quality of life, perception of optimal ageing, social support, carer stress, healthcare satisfaction, visits, comprehensiveness, patient empowerment, patient-centeredness, physical activity, falls; primary care, hospital and emergency department visits for any reason | Health TAPESTRY did not improve goal attainment and patient-reported outcomes or experiences, but increased the number of primary care visits for older adults, reduced the odds of 1 or more hospitalizations, and contributed to a shift from reactive to proactive and preventive care for patients. |
| Vestjens, Lotte et al.(33)  (2019)  Netherlands | Quasi-experimental study | Community | Frail elderly | 232 | 232 | GPs, practice nurses, home care nurses or geriatric nurses, geriatricians, physiotherapists, case managers | EMR, GP information systems, chain Information | 12 months  1. Select and screen patients for active frailty; 2. Report needs and problems in multiple domains based on the SFSPC model; 3. Provide information to GPs and geriatric care physicians for screening and problem analysis; 4. Discuss assessment results for older adults in multidisciplinary consultations; 5. Create individualized care plans (lifestyle interventions, self-management measures, multidisciplinary follow-up and assessment plans); 6. Medication Review (GP, geriatrician or pharmacist to review medication use at least annually); 7. Multidisciplinary follow-up. | Cost utility(EQ-5D-3L), subjective well-being (Social Productive Functioning Tools for Levels of Well-being (SPF-ILs)), healthcare utilization, cost | Active integrated care did not improve the quality of life of frail older people.  The intervention group had a higher average total cost, was not cost effective and had a better sense of well-being in the control group. |
| Barker, Anna et al.(21)  (2019)  Australia | RCT | Home & Hospital | Older adults | 231 | 217 | 3 physiotherapists, 2 occupational therapists, 1 nurse and 1 nutritionist | Electronic assessment tools (Ipad), telephone | 6 months  1.Home-based assessment of fall risk factors; 2. Participants are provided with four risk factor-specific educational flyer modules (strength, vision, sleep, bone) with evidence-based risk factor management information; 3. Participants are encouraged to select one or more relevant modules and develop individualized goals and action plans for each module; 4. Clinicians identify and address issues that prevent participants from implementing the program issues that prevent participants from implementing the plan and provide additional education on risk factor management; 5. Provide participants with 2 and more telephone support calls to review progress on goals and action plans; 6. Healthcare providers communicate and link to available community services to meet participants' goals. | Primary outcome: falls and fall injuries.  Secondary outcome: ED presentation, hospitalization, fracture, death.  Falls risk status, falls self-efficacy and health-related quality of life (EQ-5D-5L) | The RESPOND falls prevention programmer improves the prognosis of emergency patients and reduces falls and fractures, but does not reduce fall injuries in older people.  There was no change in hospital admission, emergency presentation, death, fall risk, fall outcomes or quality of life. |
| Uittenbroek, R J et al.(46)  (2018) Spoorenberg, Sophie L W et al.(18)  (2018)  Uittenbroek, Ronald J et al.(19)  (2017)  Netherlands | RCT | General Practitioner Clinic | Older adults | 709 | 747 | 1 GP, 1 elderly care doctor, 2 case managers (social workers (frailty) or district nurses (complex care needs)) | Clinical Information System, Electronic Elderly Record System | 12 months  1. Assessment of the complexity and vulnerability of older adults' care needs (categorising older adults as having robust, frail and complex care needs); 2. Development of a care and support plan by the case manager in consultation with the participant (robust: self-management support and prevention plan; frail and complex care needs: personal care and support plan); 3. Setting of health goals and selection of appropriate actions to be approved by team members and participants and then 4. Case managers monitor the status of participants and the implementation of care and support plans; 5. Embrace community meetings are held regularly to provide health-related information; 6. Care and support plans are regularly evaluated and updated and adjusted as necessary. | EQ-5D-3L and Visual Analogue Scale, INTERMED Self-Assessment of the Elderly, Groningen Frailty Index, Katz-15, Groningen Well-Being Index and two quality of life questions, Self-Management Competence Scale and Partners in Health for Older adults Scale (PIH-OA), Patient Assessment of Integrated Elderly Care (PAIEC) Scale, Cost-effectiveness (EQ-5D-3L) | Embrace personal care and support offset the decline in physical, cognitive and social functioning associated with ageing and improved the quality of care.  The total average cost of Embrace was higher and differences in health-related outcomes were small and not statistically significant. |
| Ruikes, FGH et al.(35)  (2018) Ruikes, Franca G H et al.(34)  (2018)  Ruikes, F G et al.(36)  (2016)  Netherlands | Cluster non-randomised controlled trial | General Practitioner Clinic | Frail elderly | 249 | 287 | GP, Practice Nurse and/or Community Nurse, Elderly Care Practitioner (ECP), Social Worker, Case Manager (Nurse or Social Worker), Pharmacist | Health and Wellbeing Information Portal (ZWIP), EHR | 12 months  1. Multidisciplinary team meetings: 1 every 4-8 weeks, with virtual communication among team members based on ZWIP; 2. Individualized care plans for participants based on individual health-related goals and needs (physical, functional, psychological, social, and communicative domains) assessed by EASY-Care TOS, stored on an informational website, and revised at least every 6 months; 3. Assignment of each participant to 1 case manager for care planning and coordination, patient goal setting and self-management, and caregiver support; 4. 1 medication review per year for participants using ≥5 chronic prescription medications; 5. Multidisciplinary guidelines, advance care planning guidelines, and procedural protocols (embedded in ZWIP to aid decision-making) developed for 8 common geriatric syndromes | Primary outcome: functional independence in activities of daily living (Katz-15 index)  Secondary outcome: quality of life (EQ-5D-3L), mental health and health-related social functioning (SF-36), institutionalization, hospitalization and mortality, intervention costs and healthcare utilization, implementation fidelity | The CareWell primary care programmer had no net monetary benefit and no significant impact on improving older adults' activity functioning, quality of life, mental health, institutionalization, hospitalization and mortality. |
| Di Pollina, Laura et al.(47)  (2017)  Switzerland | Prospective controlled trial | Home | Frail and dependent older adults | 179 | 122 | Primary care physicians, nurses, CGU family intervention teams (doctors, physical and occupational therapists, psychologists, nutritionists and social workers) | Telephone (medical call service) | 5 - 41 months  1. Home assessment by the community geriatric unit (cognition, mood, functional status, IADLs, gait and semi-tandem standing, nutrition, pain, medication and adherence); 2. Primary care physician and care team make recommendations based on the assessment; 3. Community geriatric team and care team meet to discuss complex issues; 4. Participants and care team contact primary care physician based on written instructions, or contact the CGU, which provides a 24-hour, 7-day-a-week medical call service. | Primary outcome: number of hospitalizations  Secondary outcome: cause of hospitalization, number and cause of ED visits, institutionalization, death and place of death | Integrated care significantly reduced unnecessary hospital admissions, emergency visits and allows more patients to die at home.  Improved coordination and access to care for frail and dependent older people. |
| Chan, D C D et al.(22)  (2017)  Taiwan, China | RCT | Community Hospital | Frailty and sarcopenia elderly | 146 | 143 | Nurse, sports specialist | Telephone, multimedia health education materials | 12 months  1. Low Level Care (LLC): receive a 2-hour educational session on frailty, muscle loss, coping strategies, nutrition and learning an exercise programmer demonstration (first 1h: introduction to the exercise programmer; second 1h: practice of the programmer with an exercise specialist); distribute multimedia educational materials as a reference for home practice; follow up telephone visits to participants every 2 months.  2. High Level Care (HLC): 6 on-site problem-solving sessions and 48 exercise sessions on top of the LLC, with brief nutritional advice during exercise. | Primary outcome: frailty in cardiovascular health  Secondary outcome: change in individual frailty and indicators of sarcopenia (5m walk time, dominant hand grip strength, timed ascent, walk test, left leg standing time) | Integrated care improved frailty and muscle loss in older people in the community, and high intensity training improved intensity for people at high risk and high motivation. |
| Looman, WM et al.(37)  (2016) Looman, W M et al.(38)  (2016)  Netherlands | Quasi-experimental study | General Practitioner Clinic | Frail elderly | 249 | 254 | GPs, case managers (single-needs older people: geriatric nurses; multiple or complex-needs older people: second-line geriatric care specialists), community nurses, other professionals (hospital geriatricians, nursing home doctors, physiotherapists, social workers or psychologists) | Patient file sharing system, telephone | 12 months  1. Frailty screening of older people (older adults with a Groningen Frailty Index GFI ≥4 are assigned to a case manager); 2. Single point of entry (primary care practice); 3. Evidence-based comprehensive needs assessment; 4. Development of multidisciplinary individualized service plans; 5. Case management (ensuring access to appropriate services and planning, coordination and delivery of monitored care); 6. Multidisciplinary team consultation and meetings; 7. Protocol-led care assignment; 8. Steering group of geriatric care network partners, municipalities, social care and welfare agencies responsible for planning and implementing interventions; 9. Task specialization and delegation; 10. Chained computerized system (allowing professionals to access and adapt care plans for specific frail older patients). | Cost-effectiveness (EQ-5D)  Costs: health care costs, intervention costs and informal health care costs (volume of care * cost price), Health outcome(SF-36), Functional capacity(Katz-15), Quality of life(SF-36, EQ-5D, ICECAP) | WICM has a positive impact on love and friendship and a moderate positive impact on general quality of life, but no significant impact on the health status of older people.  WICM is not cost effective and the cost per quality-adjusted life year is high. |
| Looman, W M et al.(39)  (2014)  Netherlands | Ibid | Ibid | Ibid | 224 | 222 | Ibid | Ibid | 3 months  Ibid | Health outcomes, functional capacity, quality of life (as above)  Health care utilization, satisfaction with care (Consumer Quality Index) | WICM had a small short-term effect and significantly improved the attachment dimension of quality of life, but had no significant effect on health status.  The most commonly used types of care are home care, alarm systems and meals on wheels. |
| Bakker, F C et al.(45)  (2014)  Netherlands | Pre- and post-control experiment | Hospital | Frail elderly | 195 | 91 | CareWell team (1 geriatric nurse and 1 geriatrician), nurses, doctors, volunteers | Clinician and patient information systems, nurse information systems | 3 months  1. Initial frailty screening by nurses on admission; 2. Clinical judgement of frailty by geriatric nurses; 3. Critical assessment by geriatricians from patient medical information and medication use; 4. CareWell programmer: volunteer support, medication, aftercare and goal attainment; 5. Follow-up during admission and update of CareWell programmer at discharge; 6. Surrogate medical records 7. Comprehensive geriatric assessment: guides the development of individual coordinated and integrated care plans for highly frail patients; 8. Multidisciplinary meetings: adapts medical and nursing policies to the individual needs and desires of frail elderly patients; 9. Cognitive and physical activity stimulation by trained volunteers; 10. Intervention-related education and job coaching for nurses and physicians. | Primary outcome: occurrence of hospital-acquired delirium during hospitalization, cognitive and physical functioning (MMSE), Groningen Activity Restriction Scale (GARS)  Secondary outcome: ADL, readmission and caregiver burden, morbidity burden | Mean ADLs of older people improved between discharge and follow-up.  Decreased caregiver burden 3 months post-discharge. |
| Boult, C et al.(23)  (2013)  Boyd, C M et al.(27)  (2010)  Boult, Chad et al.(26)  (2008)  USA | cRCT | General Practitioner Clinic | Chronically ill older adults (high risk of health care resource use) | 419 | 485 | Guided Care Nurse (GCN), Primary Care Doctor | Telephone, EHR | 1.5 - 3 years  1. Home assessment of patients and primary caregivers (medical, functional, cognitive, emotional, psychosocial, nutritional and environmental conditions) to determine highest priorities for optimizing health and quality of life; 2. Develop evidence-based care plans based on patient preferences, priorities and intentions; 3. Promote patient self-management (attend 15h/6 sessions); 4. Monitor patients' conditions by monthly phone calls and promptly discuss problems with primary care physician to discuss and take appropriate action; 5. Coach patients in practicing healthy behaviours; 6. Coordinate patient transitions between care settings and providers; 7. Educate and support caregivers with individual and group assistance from the GCN; 8. Recommend accessible community resources for patients and caregivers. | Health status (HCC score), functional health (SF-36), quality of health care, Primary Care Assessment Survey, health care utilization (health insurance claims), mortality, care satisfaction | Guided care improved goal setting, coordination and decision support for the complex health care needed by older people with multiple conditions, reduced the use of home care, improved patient problem-solving skills and primary care provider satisfaction with the care of older people with multiple conditions, but did not appear to improve their functional health. |
| Boyd, C M et al.(41)  (2008)  Sylvia, M L et al.(40)  (2008)  USA | Ibid | Ibid | Ibid | 75 | 75 | Ibid | Ibid | 6 months  Assessment, planning, monitoring, coaching, coordination of transition and access to community resources (chronic disease self-management programs, formal education for caregivers and support programs are excluded) | Primary care assessment, medical services expenditure, hospitalization, days in hospital and emergency visits | Guided care appears to improve the quality of the primary care experience for older people at high risk of chronic disease and reduce insurance expenditure for older people at risk. |
| Boorsma, M et al.(24)  (2011)  Netherlands | cRCT | Residential Care Facility | Older adults with physical or cognitive disabilities | 139 | 201 | Nurse assistant, family doctor, consultant (geriatrician or psychologist) | Electronic Geriatric Assessment Tool (a web-based long-term care facility version of the Resident Assessment Tool) | 6 months  1. Multidimensional geriatric assessment (functional health and care needs) for all residents every three months; 2. Discussion of assessment results and care priorities with family doctors, older people and their families to develop individualized care plans; 3. Multidisciplinary team meetings (for residents with complex care needs: at least twice a year); 4. Consultation with a geriatrician or psychologist for frail older adults with complex medical problems; 5. Adjustment of care plans every three months based on risk assessment reports for older adults. | Primary outcome: quality of care, quality of life (SF-12), cost-effectiveness (quality-adjusted life years)  Secondary outcome: quality of care indicators, activities of daily living (Groningen Activity Limitation Scale), resident perspective on quality of care (QUOTE-Elderly-16), number of hospital admissions, mortality | Integrated multidisciplinary care improves the quality of care for older adults in residential care facilities.  Functional capacity, number of hospital admissions and health-related quality of life remained comparable between the two groups.  Fewer residents in the intervention group died than in the control group. |
| Hébert, R et al.(42)  (2010)  Canada | Quasi-experimental study | Home | Frail elderly (risk of reduced function) | 773 | 728 | Case managers (nurses, social workers or other health professionals, etc.), primary care physicians, health care professionals (medical specialists, physiotherapists, occupational therapists, speech therapists) | Telephone, computerized clinical icons (SMAF-based case-mix classification system, SIGG information system for geriatrics) | 4 years  1. Coordination of decision makers and managers at regional and local levels; 2. Single entry point: mechanism for providing services in the area for frail older adults (screening callers (older adults with disabilities), disability status assessment (positive), referral to case managers); 3. Single assessment tool combined with a mixed case management system; 4. Case management (case managers work with primary care physicians to implement assessments); 5. Case manager-led, intervention plans developed at multidisciplinary team meetings and reviewed regularly; 6. Computerized clinical charting (allows for communication between agencies and clinicians). | Primary outcome: Decline in functioning and hospital utilization (A&E and inpatient)  Secondary outcome: disability, unmet need, service satisfaction, patient empowerment, caregiver burden, health and social service utilization, mortality | The PRISMA model resulted in a lower prevalence of reduced functioning, almost half the proportion of people with unmet needs and significantly higher satisfaction and empowerment rates than the control group.  The number of emergency room and hospital admissions was lower than expected. |
| Tourigny, André et al.(44)  (2004)  Canada | Quasi-experimental study | Community | Frail elderly | 210 | 272 | Case managers, health care professionals, primary care physicians | Computerized Clinical Charting (Geriatric Information System) | 3 years  1. Intersectoral coordination at strategic, tactical and clinical levels (strategic: formation of joint management committees to agree on policy and direction, resource allocation; tactical: joint section leaders, case managers and ISD network for elderly detection; clinical: implementation of case management); 2. Single entry point; 3. Single patient assessment tool (physical and mental health, social aspects and functional autonomy); 4. Case management; 5. Development of personalized service plans; 6. Computerized clinical charting (exchange of clinical information, sharing) | Hospitalization rates, functional autonomy, caregiver burden, mortality, service use | The ISD network reduced hospital admissions and hospital intentions, eased caregiver burden, and delayed functional decline and deterioration in frail older adults in the short term.  A smaller proportion of the study group returned to emergency care within 10 days of the first visit, but there was no impact in terms of service utilization, emergency care, hospitalization or medication use. |
| Mary D. Naylor et al.(28)  (2004)  USA | RCT | Hospital to home transition | Older adults hospitalized with heart failure | 121 | 118 | 3 Advanced Practice Nurses (APN), doctors | Telephone (APN follow-up), cassette and tape recorder audio (health education materials) | 3 months  1.During hospitalization: comprehensive patient assessment, identification of patient and caregiver goals, development and implementation of individualized care plans by the APN in collaboration with physicians and other providers (evidence-based protocols guided by guidelines), provision of educational and behavioural strategies that currently meet the needs of patients and their caregivers, arrangement of home care services as required (prevention of functional decline and simplification of medication regimens), coordination of essential in collaboration with discharge planners ordering of medical supplies. 2.Discharge home: targeted assessment to identify changes in the patient's health status and work with the doctor to implement strategies to prevent the onset or minimise the impact of symptoms. | Time to first re-hospitalization or death, number of re-hospitalizations, quality of life, functional status, costs and satisfaction with care | Comprehensive transitional care interventions can extend the time between discharge and readmission or death, reduce the total number of readmissions and lower healthcare costs. |
