# Supplementary material for: Effectiveness of information and communication technology-based integrated care for older adults: a systematic review and meta-analysis
Source: Front Public Health. 2024 Jan 5;11:1276574. doi: 10.3389/fpubh.2023.1276574 (PMC10797014; doi:10.3389/fpubh.2023.1276574)
Supplement: Supplementary file 2 [file Table_2.DOCX]

# Supplementary materials S2 – Subject Headings /Entry Terms

| **Subject Headings** | **Entry Terms** |
| --- | --- |
| Aged | old adults |
|  | old people  senior citizen  elderly  elder  geriatric |
| Information Technology | Information Technologies |
|  | Technology, Information |
|  | information and communication technology  ICT  digital  tele*  internet  mobile  cloud  eHealth |
|  | virtual care |
| Delivery of Health Care, Integrated | Integrated Health Care Systems |
|  | Integrated Delivery Systems |
|  | Delivery System, Integrated |
|  | Delivery Systems, Integrated |
|  | Integrated Delivery System |
|  | System, Integrated Delivery |
|  | Systems, Integrated Delivery |
|  | integrated care  coordinated care  comprehensive care  seamless care  transmural care  multidisciplinary care  holistic care  joint care  person- centred care |
|  | interprofessional care |
|  | team-based care |
| Randomized Controlled Trials as Topic | Clinical Trials, Randomized |
|  | Trials, Randomized Clinical |
|  | Controlled Clinical Trials, Randomized |
|  | randomised controlled trial |
|  | RCT |
| Non-Randomized Controlled Trials as Topic | Non Randomized Controlled Trials as Topic |
|  | Controlled Clinical Trials, Non-Randomized |
|  | Controlled Clinical Trials, Non Randomized |
|  | Quasi-Experimental Studies |
|  | Quasi Experimental Studies  Quasi-Experimental Study |
|  | Studies, Quasi-Experimental |
|  | Study, Quasi-Experimental |
|  | Clinical Trials, Nonrandomized |
|  | Clinical Trial, Nonrandomized |
|  | Nonrandomized Clinical Trial |
|  | Nonrandomized Clinical Trials |
|  | Trial, Nonrandomized Clinical |
|  | Trials, Nonrandomized Clinical |
|  | Controlled Clinical Trials, Nonrandomized |
|  | Clinical Trials, Non-Randomized |
|  | Clinical Trial, Non-Randomized |
|  | Clinical Trials, Non Randomized |
|  | Non-Randomized Clinical Trial |
|  | Non-Randomized Clinical Trials |
|  | Trial, Non-Randomized Clinical |
|  | Trials, Non-Randomized Clinical |
|  | Nonrandomized Controlled Trials as Topic |
