# Supplementary material for: Effectiveness of information and communication technology-based integrated care for older adults: a systematic review and meta-analysis
Source: Front Public Health. 2024 Jan 5;11:1276574. doi: 10.3389/fpubh.2023.1276574 (PMC10797014; doi:10.3389/fpubh.2023.1276574)
Supplement: Supplementary file 3 [file Table_3.DOCX]

# Supplementary materials S3 – Search Strategies for each database

**PubMed**

| **Search** | **Query** |
| --- | --- |
| #5 | #1 AND #2 AND #3 AND #4 |
| #4 | "randomized controlled trials as topic"[MeSH Terms] OR "non randomized controlled trials as topic"[MeSH Terms] OR "clinical trials randomized"[Title/Abstract] OR "trials randomized clinical"[Title/Abstract] OR "controlled clinical trials randomized"[Title/Abstract] OR "randomised controlled trial"[Title/Abstract] OR "RCT"[Title/Abstract] OR "non randomized controlled trials as topic"[Title/Abstract] OR "controlled clinical trials non randomized"[Title/Abstract] OR "controlled clinical trials non randomized"[Title/Abstract] OR "quasi experimental studies"[Title/Abstract] OR "quasi experimental studies"[Title/Abstract] OR "quasi experimental study"[Title/Abstract] OR "studies quasi experimental"[Title/Abstract] OR "study quasi experimental"[Title/Abstract] OR "clinical trials nonrandomized"[Title/Abstract] OR "clinical trial nonrandomized"[Title/Abstract] OR "nonrandomized clinical trial"[Title/Abstract] OR "nonrandomized clinical trials"[Title/Abstract] OR "trial nonrandomized clinical"[Title/Abstract] OR "trials nonrandomized clinical"[Title/Abstract] OR "controlled clinical trials nonrandomized"[Title/Abstract] OR "clinical trials non randomized"[Title/Abstract] OR "clinical trial non randomized"[Title/Abstract] OR "clinical trials non randomized"[Title/Abstract] OR "non randomized clinical trial"[Title/Abstract] OR "non randomized clinical trials"[Title/Abstract] OR "trial non randomized clinical"[Title/Abstract] OR "trials non randomized clinical"[Title/Abstract] OR (("non randomized controlled trials as topic"[MeSH Terms] OR ("Non-Randomized"[All Fields] AND "Controlled"[All Fields] AND "Trials"[All Fields] AND "Topic"[All Fields]) OR "non randomized controlled trials as topic"[All Fields] OR "Nonrandomized"[All Fields]) AND "controlled trials as topic"[Title/Abstract]) |
| #3 | "delivery of health care, integrated"[MeSH Terms] OR "integrated health care systems"[Title/Abstract] OR "integrated delivery systems"[Title/Abstract] OR "delivery system integrated"[Title/Abstract] OR "delivery systems integrated"[Title/Abstract] OR "integrated delivery system"[Title/Abstract] OR (("drug delivery systems"[MeSH Terms] OR ("drug"[All Fields] AND "Delivery"[All Fields] AND "Systems"[All Fields]) OR "drug delivery systems"[All Fields] OR "System"[All Fields] OR "system s"[All Fields] OR "Systems"[All Fields]) AND "integrated delivery"[Title/Abstract]) OR "systems integrated delivery"[Title/Abstract] OR "integrated care"[Title/Abstract] OR "coordinated care"[Title/Abstract] OR "comprehensive care"[Title/Abstract] OR "seamless care"[Title/Abstract] OR "transmural care"[Title/Abstract] OR "multidisciplinary care"[Title/Abstract] OR "holistic care"[Title/Abstract] OR "joint care"[Title/Abstract] OR "person centred care"[Title/Abstract] OR "interprofessional care"[Title/Abstract] OR "team based care"[Title/Abstract] |
| #2 | "information technology"[MeSH Terms] OR "information technologies"[Title/Abstract] OR "technology information"[Title/Abstract] OR ("Information"[Title/Abstract] AND "communication technology"[Title/Abstract]) OR "ICT"[Title/Abstract] OR "digital"[Title/Abstract] OR "tele*"[Title/Abstract] OR "internet"[Title/Abstract] OR "mobile"[Title/Abstract] OR "cloud"[Title/Abstract] OR "eHealth"[Title/Abstract] OR "virtual care"[Title/Abstract] |
| #1 | "aged"[MeSH Terms] OR "old adults"[Title/Abstract] OR "old people"[Title/Abstract] OR "senior citizen"[Title/Abstract] OR "elderly"[Title/Abstract] OR "elder"[Title/Abstract] OR "geriatric"[Title/Abstract] |

**Web of Science**

| **Search** | **Query** |
| --- | --- |
| #5 | #1 AND #2 AND #3 AND #4 |
| #4 | Randomized Controlled Trials as Topic (主题) or Clinical Trials, Randomized (主题) or Trials, Randomized Clinical (主题) or Controlled Clinical Trials, Randomized (主题) or randomised controlled trial (主题) or RCT (主题) or Non-Randomized Controlled Trials as Topic (主题) or Non Randomized Controlled Trials as Topic (主题) or Controlled Clinical Trials, Non-Randomized (主题) or Controlled Clinical Trials, Non Randomized (主题) or Quasi-Experimental Studies (主题) or Quasi Experimental Studies (主题) or Quasi-Experimental Study (主题) or Studies, Quasi-Experimental (主题) or Study, Quasi-Experimental (主题) or Clinical Trials, Nonrandomized (主题) or Clinical Trial, Nonrandomized (主题) or Nonrandomized Clinical Trial (主题) or Nonrandomized Clinical Trials (主题) or Trial, Nonrandomized Clinical (主题) or Trials, Nonrandomized Clinical (主题) or Controlled Clinical Trials, Nonrandomized (主题) or Clinical Trials, Non-Randomized (主题) or Clinical Trial, Non-Randomized (主题) or Clinical Trials, Non Randomized (主题) or Non-Randomized Clinical Trial (主题) or Non-Randomized Clinical Trials (主题) or Trial, Non-Randomized Clinical (主题) or Trials, Non-Randomized Clinical (主题) or Nonrandomized Controlled Trials as Topic (主题) |
| #3 | Delivery of Health Care, Integrated (主题) or Integrated Health Care Systems (主题) or Integrated Delivery Systems (主题) or Delivery System, Integrated (主题) or Delivery Systems, Integrated (主题) or Integrated Delivery System (主题) or System, Integrated Delivery (主题) or Systems, Integrated Delivery (主题) or integrated care (主题) or coordinated care (主题) or comprehensive care (主题) or seamless care (主题) or transmural care (主题) or multidisciplinary care (主题) or holistic care (主题) or joint care (主题) or person- centred care (主题) or interprofessional care (主题) or team-based care (主题) |
| #2 | Information Technology (主题) or Information Technologies (主题) or Technology, Information (主题) or information and communication technology (主题) or ICT (主题) or digital (主题) or tele* (主题) or internet (主题) or mobile (主题) or cloud (主题) or eHealth (主题) or virtual care (主题) |
| #1 | Aged (主题) or old adults (主题) or old people (主题) or senior citizen (主题) or elderly (主题) or elder (主题) or geriatric (主题) |

**Scopus**

| **Search** | **Query** |
| --- | --- |
| #5 | #1 AND #2 AND #3 AND #4 |
| #4 | ( TITLE-ABS-KEY ( randomized AND controlled AND trials AND as AND topic ) OR TITLE-ABS-KEY ( clinical AND trials, AND randomized ) OR TITLE-ABS-KEY ( trials, AND randomized AND clinical ) OR TITLE-ABS-KEY ( controlled AND clinical AND trials, AND randomized ) OR TITLE-ABS-KEY ( randomised AND controlled AND trial ) OR TITLE-ABS-KEY ( rct ) OR TITLE-ABS-KEY ( non-randomized AND controlled AND trials AND as AND topic ) OR TITLE-ABS-KEY ( non AND randomized AND controlled AND trials AND as AND topic ) OR TITLE-ABS-KEY ( controlled AND clinical AND trials, AND non-randomized ) OR TITLE-ABS-KEY ( controlled AND clinical AND trials, AND non AND randomized ) OR TITLE-ABS-KEY ( quasi-experimental AND studies ) OR TITLE-ABS-KEY ( quasi AND experimental AND studies ) OR TITLE-ABS-KEY ( quasi-experimental AND study ) OR TITLE-ABS-KEY ( studies, AND quasi-experimental ) OR TITLE-ABS-KEY ( study, AND quasi-experimental ) OR TITLE-ABS-KEY ( clinical AND trials, AND nonrandomized ) OR TITLE-ABS-KEY ( clinical AND trial, AND nonrandomized ) OR TITLE-ABS-KEY ( nonrandomized AND clinical AND trial ) OR TITLE-ABS-KEY ( nonrandomized AND clinical AND trials ) OR TITLE-ABS-KEY ( trial, AND nonrandomized AND clinical ) OR TITLE-ABS-KEY ( trials, AND nonrandomized AND clinical ) OR TITLE-ABS-KEY ( controlled AND clinical AND trials, AND nonrandomized ) OR TITLE-ABS-KEY ( clinical AND trials, AND non-randomized ) OR TITLE-ABS-KEY ( clinical AND trial, AND non-randomized ) OR TITLE-ABS-KEY ( clinical AND trials, AND non AND randomized ) OR TITLE-ABS-KEY ( non-randomized AND clinical AND trial ) OR TITLE-ABS-KEY ( non-randomized AND clinical AND trials ) OR TITLE-ABS-KEY ( trial, AND non-randomized AND clinical ) OR TITLE-ABS-KEY ( trials, AND non-randomized AND clinical ) OR TITLE-ABS-KEY ( nonrandomized AND controlled AND trials AND as AND topic ) ) |
| #3 | ( TITLE-ABS-KEY ( delivery AND of AND health AND care, AND integrated ) OR TITLE-ABS-KEY ( integrated AND health AND care AND systems ) OR TITLE-ABS-KEY ( integrated AND delivery AND systems ) OR TITLE-ABS-KEY ( delivery AND system, AND integrated ) OR TITLE-ABS-KEY ( delivery AND systems, AND integrated ) OR TITLE-ABS-KEY ( integrated AND delivery AND system ) OR TITLE-ABS-KEY ( system, AND integrated AND delivery ) OR TITLE-ABS-KEY ( systems, AND integrated AND delivery ) OR TITLE-ABS-KEY ( integrated AND care ) OR TITLE-ABS-KEY ( coordinated AND care ) OR TITLE-ABS-KEY ( comprehensive AND care ) OR TITLE-ABS-KEY ( seamless AND care ) OR TITLE-ABS-KEY ( transmural AND care ) OR TITLE-ABS-KEY ( multidisciplinary AND care ) OR TITLE-ABS-KEY ( holistic AND care ) OR TITLE-ABS-KEY ( joint AND care ) OR TITLE-ABS-KEY ( person- AND centred AND care ) OR TITLE-ABS-KEY ( interprofessional AND care ) OR TITLE-ABS-KEY ( team-based AND care ) ) |
| #2 | ( TITLE-ABS-KEY ( information AND technology ) OR TITLE-ABS-KEY ( information AND technologies ) OR TITLE-ABS-KEY ( technology, AND information ) OR TITLE-ABS-KEY ( information AND communication AND technology ) OR TITLE-ABS-KEY ( ict ) OR TITLE-ABS-KEY ( digital ) OR TITLE-ABS-KEY ( tele* ) OR TITLE-ABS-KEY ( internet ) OR TITLE-ABS-KEY ( mobile ) OR TITLE-ABS-KEY ( cloud ) OR TITLE-ABS-KEY ( ehealth ) OR TITLE-ABS-KEY ( virtual AND care ) ) |
| #1 | ( TITLE-ABS-KEY ( aged ) OR TITLE-ABS-KEY ( old AND adults ) OR TITLE-ABS-KEY ( old AND people ) OR TITLE-ABS-KEY ( senior AND citizen ) OR TITLE-ABS-KEY ( elderly ) OR TITLE-ABS-KEY ( elder ) OR TITLE-ABS-KEY ( geriatric ) ) |

**MEDLINE**

| **Search** | **Query** |
| --- | --- |
| #5 | #1 AND #2 AND #3 AND #4 |
| #4 | Randomized Controlled Trials as Topic (MeSH 主题词) or Non-Randomized Controlled Trials as Topic (MeSH 主题词) or Clinical Trials, Randomized (主题) or Trials, Randomized Clinical (主题) or Controlled Clinical Trials, Randomized (主题) or randomised controlled trial (主题) or RCT (主题) or Non Randomized Controlled Trials as Topic (主题) or Controlled Clinical Trials, Non-Randomized (主题) or Controlled Clinical Trials, Non Randomized (主题) or Quasi-Experimental Studies (主题) or Quasi Experimental Studies (主题) or Quasi-Experimental Study (主题) or Studies, Quasi-Experimental (主题) or Study, Quasi-Experimental (主题) or Clinical Trials, Nonrandomized (主题) or Clinical Trial, Nonrandomized (主题) or Nonrandomized Clinical Trial (主题) or Nonrandomized Clinical Trials (主题) or Trial, Nonrandomized Clinical (主题) or Trials, Nonrandomized Clinical (主题) or Controlled Clinical Trials, Nonrandomized (主题) or Clinical Trials, Non-Randomized (主题) or Clinical Trial, Non-Randomized (主题) or Clinical Trials, Non Randomized (主题) or Non-Randomized Clinical Trial (主题) or Non-Randomized Clinical Trials (主题) or Trial, Non-Randomized Clinical (主题) or Trials, Non-Randomized Clinical (主题) or Nonrandomized Controlled Trials as Topic (主题) |
| #3 | Delivery of Health Care, Integrated (MeSH 主题词) or Integrated Health Care Systems (主题) or Integrated Delivery Systems (主题) or Delivery System, Integrated (主题) or Delivery Systems, Integrated (主题) or Integrated Delivery System (主题) or System, Integrated Delivery (主题) or Systems, Integrated Delivery (主题) or integrated care (主题) or coordinated care (主题) or comprehensive care (主题) or seamless care (主题) or transmural care (主题) or multidisciplinary care (主题) or holistic care (主题) or joint care (主题) or person- centred care (主题) or interprofessional care (主题) or team-based care (主题) |
| #2 | Information Technology (MeSH 主题词) or Information Technologies (主题) or Technology, Information (主题) or information and communication technology (主题) or ICT (主题) or digital (主题) or tele* (主题) or internet (主题) or mobile (主题) or cloud (主题) or eHealth (主题) or virtual care (主题) |
| #1 | Aged (MeSH 主题词) or old adults (主题) or old people (主题) or senior citizen (主题) or elderly (主题) or elder (主题) or geriatric (主题) |

**EBSCO**

| **Search** | **Query** |
| --- | --- |
| #5 | #1 AND #2 AND #3 AND #4 |
| #4 | SU Randomized Controlled Trials as Topic OR SU Clinical Trials, Randomized OR SU Trials, Randomized Clinical OR SU Controlled Clinical Trials, Randomized OR SU randomised controlled trial OR SU RCT OR SU Non-Randomized Controlled Trials as Topic OR SU Non Randomized Controlled Trials as Topic OR SU Controlled Clinical Trials, Non-Randomized OR SU Controlled Clinical Trials, Non Randomized OR SU Quasi-Experimental Studies OR SU Quasi Experimental Studies OR SU Quasi-Experimental Study OR SU Studies, Quasi-Experimental OR SU Study, Quasi-Experimental OR SU Clinical Trials, Nonrandomized OR SU Clinical Trial, Nonrandomized OR SU Nonrandomized Clinical Trial OR SU Nonrandomized Clinical Trials OR SU Trial, Nonrandomized Clinical OR SU Trials, Nonrandomized Clinical OR SU Controlled Clinical Trials, Nonrandomized OR SU Clinical Trials, Non-Randomized OR SU Clinical Trial, Non-Randomized OR SU Clinical Trials, Non Randomized OR SU Non-Randomized Clinical Trial OR SU Non-Randomized Clinical Trials OR SU Trial, Non-Randomized Clinical OR SU Trials, Non-Randomized Clinical OR SU Nonrandomized Controlled Trials as Topic |
| #3 | SU Delivery of Health Care, Integrated OR SU Integrated Health Care Systems OR SU Integrated Delivery Systems OR SU Delivery System, Integrated OR SU Delivery Systems, Integrated OR SU Integrated Delivery System OR SU System, Integrated Delivery OR SU Systems, Integrated Delivery OR SU integrated care OR SU coordinated care OR SU comprehensive care OR SU seamless care OR SU transmural care OR SU multidisciplinary care OR SU holistic care OR SU joint care OR SU person- centred care OR SU interprofessional care OR SU team-based care |
| #2 | SU Information Technology OR SU Information Technologies OR SU Technology, Information OR SU ( information and communication technology ) OR SU ICT OR SU digital OR SU tele* OR SU internet OR SU mobile OR SU cloud OR SU eHealth OR SU virtual care |
| #1 | SU Aged OR SU old adults OR SU old people OR SU senior citizen OR SU elderly OR SU elder OR SU geriatric |

**CINAHL Plus with Full Text**

| **Search** | **Query** |
| --- | --- |
| #5 | #1 AND #2 AND #3 AND #4 |
| #4 | MH Randomized Controlled Trials as Topic OR MH Non-Randomized Controlled Trials as Topic OR SU Clinical Trials, Randomized OR SU Trials, Randomized Clinical OR SU Controlled Clinical Trials, Randomized OR SU randomised controlled trial OR SU RCT OR SU Non Randomized Controlled Trials as Topic OR SU Controlled Clinical Trials, Non-Randomized OR SU Controlled Clinical Trials, Non Randomized OR SU Quasi-Experimental Studies OR SU Quasi Experimental Studies OR SU Quasi-Experimental Study OR SU Studies, Quasi-Experimental OR SU Study, Quasi-Experimental OR SU Clinical Trials, Nonrandomized OR SU Clinical Trial, Nonrandomized OR SU Nonrandomized Clinical Trial OR SU Nonrandomized Clinical Trials OR SU Trial, Nonrandomized Clinical OR SU Trials, Nonrandomized Clinical OR SU Controlled Clinical Trials, Nonrandomized OR SU Clinical Trials, Non-Randomized OR SU Clinical Trial, Non-Randomized OR SU Clinical Trials, Non Randomized OR SU Non-Randomized Clinical Trial OR SU Non-Randomized Clinical Trials OR SU Trial, Non-Randomized Clinical OR SU Trials, Non-Randomized Clinical OR SU Nonrandomized Controlled Trials as Topic |
| #3 | MH Delivery of Health Care, Integrated OR SU Integrated Health Care Systems OR SU Integrated Delivery Systems OR SU Delivery System, Integrated OR SU Delivery Systems, Integrated OR SU Integrated Delivery System OR SU System, Integrated Delivery OR SU Systems, Integrated Delivery OR SU integrated care OR SU coordinated care OR SU comprehensive care OR SU seamless care OR SU transmural care OR SU multidisciplinary care OR SU holistic care OR SU joint care OR SU person- centred care OR SU interprofessional care OR SU team-based care |
| #2 | MH Information Technology OR SU Information Technologies OR SU Technology, Information OR SU ( information and communication technology ) OR SU ICT OR SU digital OR SU tele* OR SU internet OR SU mobile OR SU cloud OR SU eHealth OR SU virtual care |
| #1 | MH Aged OR SU old adults OR SU old people OR SU senior citizen OR SU elderly OR SU elder OR SU geriatric |

**Embase**

| **Search** | **Query** |
| --- | --- |
| #14 | #3 AND #6 AND #9 AND #13 |
| #13 | #10 OR #11 OR #12 |
| #12 | 'clinical trials, randomized':ti,ab,kw OR 'trials, randomized clinical':ti,ab,kw OR 'controlled clinical trials, randomized':ti,ab,kw OR 'randomised controlled trial':ti,ab,kw OR rct:ti,ab,kw OR 'non randomized controlled trials as topic':ti,ab,kw OR 'controlled clinical trials, non-randomized':ti,ab,kw OR 'controlled clinical trials, non randomized':ti,ab,kw OR 'quasi-experimental studies':ti,ab,kw OR 'quasi experimental studies':ti,ab,kw OR 'quasi-experimental study':ti,ab,kw OR 'studies, quasi-experimental':ti,ab,kw OR 'study, quasi-experimental':ti,ab,kw OR 'clinical trials, nonrandomized':ti,ab,kw OR 'clinical trial, nonrandomized':ti,ab,kw OR 'nonrandomized clinical trial':ti,ab,kw OR 'nonrandomized clinical trials':ti,ab,kw OR 'trial, nonrandomized clinical':ti,ab,kw OR 'trials, nonrandomized clinical':ti,ab,kw OR 'controlled clinical trials, nonrandomized':ti,ab,kw OR 'clinical trials, non-randomized':ti,ab,kw OR 'clinical trial, non-randomized':ti,ab,kw OR 'clinical trials, non randomized':ti,ab,kw OR 'non-randomized clinical trial':ti,ab,kw OR 'non-randomized clinical trials':ti,ab,kw OR 'trial, non-randomized clinical':ti,ab,kw OR 'trials, non-randomized clinical':ti,ab,kw OR 'nonrandomized controlled trials as topic':ti,ab,kw |
| #11 | 'controlled clinical trial (topic)'/exp |
| #10 | 'randomized controlled trial (topic)'/exp |
| #9 | #7 OR #8 |
| #8 | 'delivery of health care, integrated':ti,ab,kw OR 'integrated health care systems':ti,ab,kw OR 'integrated delivery systems':ti,ab,kw OR 'delivery system, integrated':ti,ab,kw OR 'delivery systems, integrated':ti,ab,kw OR 'integrated delivery system':ti,ab,kw OR 'system, integrated delivery':ti,ab,kw OR 'systems, integrated delivery':ti,ab,kw OR 'integrated care':ti,ab,kw OR 'coordinated care':ti,ab,kw OR 'comprehensive care':ti,ab,kw OR 'seamless care':ti,ab,kw OR 'transmural care':ti,ab,kw OR 'multidisciplinary care':ti,ab,kw OR 'holistic care':ti,ab,kw OR 'joint care':ti,ab,kw OR 'person- centred care':ti,ab,kw OR 'interprofessional care':ti,ab,kw OR 'team-based care':ti,ab,kw |
| #7 | 'integrated health care system'/exp |
| #6 | #4 OR #5 |
| #5 | 'information technologies':ti,ab,kw OR 'technology, information':ti,ab,kw OR (information:ti,ab,kw AND 'communication technology':ti,ab,kw) OR ict:ti,ab,kw OR digital:ti,ab,kw OR tele*:ti,ab,kw OR internet:ti,ab,kw OR mobile:ti,ab,kw OR cloud:ti,ab,kw OR ehealth:ti,ab,kw OR 'virtual care':ti,ab,kw |
| #4 | 'information technology'/exp |
| #3 | #1 OR #2 |
| #2 | 'old adults':ti,ab,kw OR 'old people':ti,ab,kw OR 'senior citizen':ti,ab,kw OR elderly:ti,ab,kw OR elder:ti,ab,kw OR geriatric:ti,ab,kw |
| #1 | 'aged'/exp |

**ProQuest**

| **Search** | **Query** |
| --- | --- |
| #5 | #1 AND #2 AND #3 AND #4 |
| #4 | su(randomized controlled trials as topic) OR su(clinical trials, randomized) OR su(trials, randomized clinical) OR su(controlled clinical trials, randomized) OR su(randomised controlled trial) OR su(RCT) OR su(non-randomized controlled trials as topic) OR su(non randomized controlled trials as topic) OR su(controlled clinical trials, non-randomized) OR su(controlled clinical trials, non randomized) OR su(quasi-experimental studies) OR su(quasi experimental studies) OR su(quasi-experimental study) OR su(studies, quasi-experimental) OR su(study, quasi-experimental) OR su(clinical trials, nonrandomized) OR su(clinical trial, nonrandomized) OR su(nonrandomized clinical trial) OR su(nonrandomized clinical trials) OR su(trial, nonrandomized clinical) OR su(trials, nonrandomized clinical) OR su(controlled clinical trials, nonrandomized) OR su(clinical trials, non-randomized) OR su(clinical trial, non-randomized) OR su(clinical trials, non randomized) OR su(non-randomized clinical trial) OR su(non-randomized clinical trials) OR su(trial, non-randomized clinical) OR su(trials, non-randomized clinical) OR su(nonrandomized controlled trials as topic) |
| #3 | su(information technology) OR su(information technologies) OR su(technology, information) OR su(information AND communication technology) OR su(ICT) OR su(digital) OR su(tele*) OR su(internet) OR su(mobile) OR su(cloud) OR su(eHealth) OR su(virtual care) |
| #2 | su(delivery of health care, integrated) OR su(integrated health care systems) OR (integrated delivery systems) OR su(delivery system, integrated) OR su(delivery systems, integrated) OR su(integrated delivery system) OR su(system, integrated delivery) OR su(systems, integrated delivery) OR su(integrated care) OR (integrated health system) OR su(coordinated care) OR su(comprehensive care) OR su(seamless care) OR su(transmural care) OR su(multidisciplinary care) OR su(holistic care) OR su(joint care) OR su(person- centred care) OR su(interprofessional care) OR su(team-based care) |
| #1 | su(Aged) OR su(old adults) OR su(old people) OR (senior citizen) OR su(elderly) OR su(elder) OR su(geriatric) |

**Cochrane**

| **Search** | **Query** |
| --- | --- |
| #15 | #3 AND #6 AND #9 AND #14 |
| #14 | #10 OR #11 OR #12 OR #113 |
| #13 | (Quasi-Experimental Study):ti,ab,kw OR (Clinical Trials, Nonrandomized):ti,ab,kw OR (Nonrandomized Clinical Trial):ti,ab,kw OR (Controlled Clinical Trials, Non-Randomized):ti,ab,kw OR (Trial, Nonrandomized Clinical):ti,ab,kw |
| #12 | (randomised controlled trial):ti,ab,kw OR (RCT):ti,ab,kw OR (Clinical Trials, Randomized):ti,ab,kw OR (Trials, Randomized Clinical):ti,ab,kw OR (Controlled Clinical Trials, Randomized):ti,ab,kw |
| #11 | MeSH descriptor: [Non-Randomized Controlled Trials as Topic] explode all trees |
| #10 | MeSH descriptor: [Randomized Controlled Trials as Topic] explode all trees |
| #9 | #7 OR #8 |
| #8 | (integrated care):ti,ab,kw OR (coordinated care):ti,ab,kw OR (multidisciplinary care):ti,ab,kw OR (person centred care):ti,ab,kw OR (team based care):ti,ab,kw |
| #7 | MeSH descriptor: [Delivery of Health Care, Integrated] explode all trees |
| #6 | #4 OR #5 |
| #5 | (information and communication technology):ti,ab,kw OR (eHealth):ti,ab,kw OR (tele*):ti,ab,kw OR (internet):ti,ab,kw OR (virtual care):ti,ab,kw |
| #4 | MeSH descriptor: [Information Technology] explode all trees |
| #3 | #1 OR #2 |
| #2 | (old adults):ti,ab,kw OR (old people):ti,ab,kw OR (senior citizen):ti,ab,kw OR (elderly):ti,ab,kw OR (geriatric):ti,ab,kw |
| #1 | MeSH descriptor: [Aged] in all MeSH products |
